# The emergence of the two cell fates and their associated switching for a negative auto-regulating gene

**DOI:** 10.1186/s12915-019-0666-0

**Published:** 2019-06-15

**Authors:** Zhenlong Jiang, Li Tian, Xiaona Fang, Kun Zhang, Qiong Liu, Qingzhe Dong, Erkang Wang, Jin Wang

**Affiliations:** 1 State Key Laboratory of Electroanalytical Chemistry, Changchun Institute of Applied Chemistry, Chinese Academy of Sciences, Changchun, Jilin, 130022 China; 2 Department of Chemistry, Physics and Applied Mathematics, State University of New York at Stony Brook, Stony Brook, New York, 11794-3400 USA; 3 College of Physics, Jilin University, Changchun, Jilin, 130012 China

**Keywords:** Gene expression, Self-repressor, Bimodality, Cell fate decision-making

## Abstract

**Background:**

Decisions in the cell that lead to its ultimate fate are important for fundamental cellular functions such as proliferation, growth, differentiation, development, and death. These cell fate decisions can be influenced by both the gene regulatory network and also environmental factors and can be modeled using simple gene feedback circuits. Negative auto-regulation is a common feedback motif in the gene circuits. It can act to reduce gene expression noise or induce oscillatory expression and is thought to lead to only one cell fate. Here, we present experimental and modeling data to suggest that a self-repressor circuit can lead to two cell fates under specific conditions.

**Results:**

We show that the introduction of inducers capable of binding and unbinding to a self-repressing gene product (protein), thus regulating the associated gene, can lead to the emergence of two cell states. We suggest that the inducers can alter the effective regulatory binding and unbinding speed of the self-repressor regulatory protein to its destination DNA without changing the gene itself. The corresponding simulation results are consistent with the experimental findings. We propose physical and quantitative explanations for the origin of the two phenotypic cell fates.

**Conclusions:**

Our results suggest a mechanism for the emergence of multiple cell fates. This may explain the heterogeneity often observed among cell states, while illustrating that altering gene regulation strength can influence cell fates and their decision-making processes without genetic changes.

**Electronic supplementary material:**

The online version of this article (10.1186/s12915-019-0666-0) contains supplementary material, which is available to authorized users.

## Background

Uncovering the origin of the phenotypes or fates of the cell and their associated switching is important for the full understanding of cell functions such as proliferation, growth, differentiation, development, and death. This remains a challenging issue in biology. It is clear that the underlying gene regulatory networks are crucial in determining the function of the cell [1–6]. Usually, phenotypes of organisms or cells can be determined and are measurable. A phenotype is often thought to be determined by the genotype in the conventional perspective [7–11]. Recently, some studies have indicated that microenvironments or epigenetics can also alter the fates of the cell or its phenotypes even with the same genotypes [12–21]. In other words, there is a possibility that apart from mutating the genes or the nodes themselves in the gene circuit, changing the underlying gene regulatory wirings among the genes or nodes in the regulatory network can alter the cell phenotypes or fates. In this study, we aim to study how altering gene regulation determines cell fates.

Negative auto-regulation is abundant: it is found in nearly 50% of the feedback loops in gene regulatory networks. It is widely believed that negative auto-regulation leads to a reduction of the gene expression noise, an increase of gene response times, an induction of possible oscillatory gene expression, and an improvement of the stability of proteins produced by the underlying gene networks [22–26]. Despite these novel findings, most experimental studies have been focused on the influences of the genetic structures themselves, rather than the environmental or the epigenetic effects on the self-repressor.

For a self-repressing system, the expression distribution is commonly more concentrated and well-distributed as a unimodal distribution [27]. Many previous investigations have reached similar conclusions, observing only one cell fate [25, 28–30]. However, these experiments were performed mostly in simple organisms such as bacteria, for which it is often assumed that the speed of regulatory protein binding/unbinding to the corresponding DNA for switching is significantly faster than the synthesis and degradation of the corresponding regulatory proteins. In fact, in most organisms, cell complexes such as the nuclei inside mammalian cells may give rise to effectively slower processes of the underlying gene regulatory binding/unbinding, due to environmental complexities such as epigenetic effects through histone modification or DNA methylation. That is, the effective rates of binding/unbinding of the regulatory proteins to the DNA can be comparable to, or even slower than, the production and degradation rate of the regulatory proteins [31]. Modeling studies [32–34] indicate that, in this case, the protein expressions of a negative feedback loop motif may not always show a simple single steady state, but instead can show two steady states, resulting in two different cell fates. Since the auto-regulation circuit involves only a single gene, it is the simplest gene regulation in vivo. We will show experimentally that this simple gene auto-regulation circuit can lead to different cell fates or phenotypes under specific conditions, rather than that of only one cell fate as is commonly expected.

## Results

### Self-repressing gene circuit and non-regulatory gene circuit

**Fig. 1:**
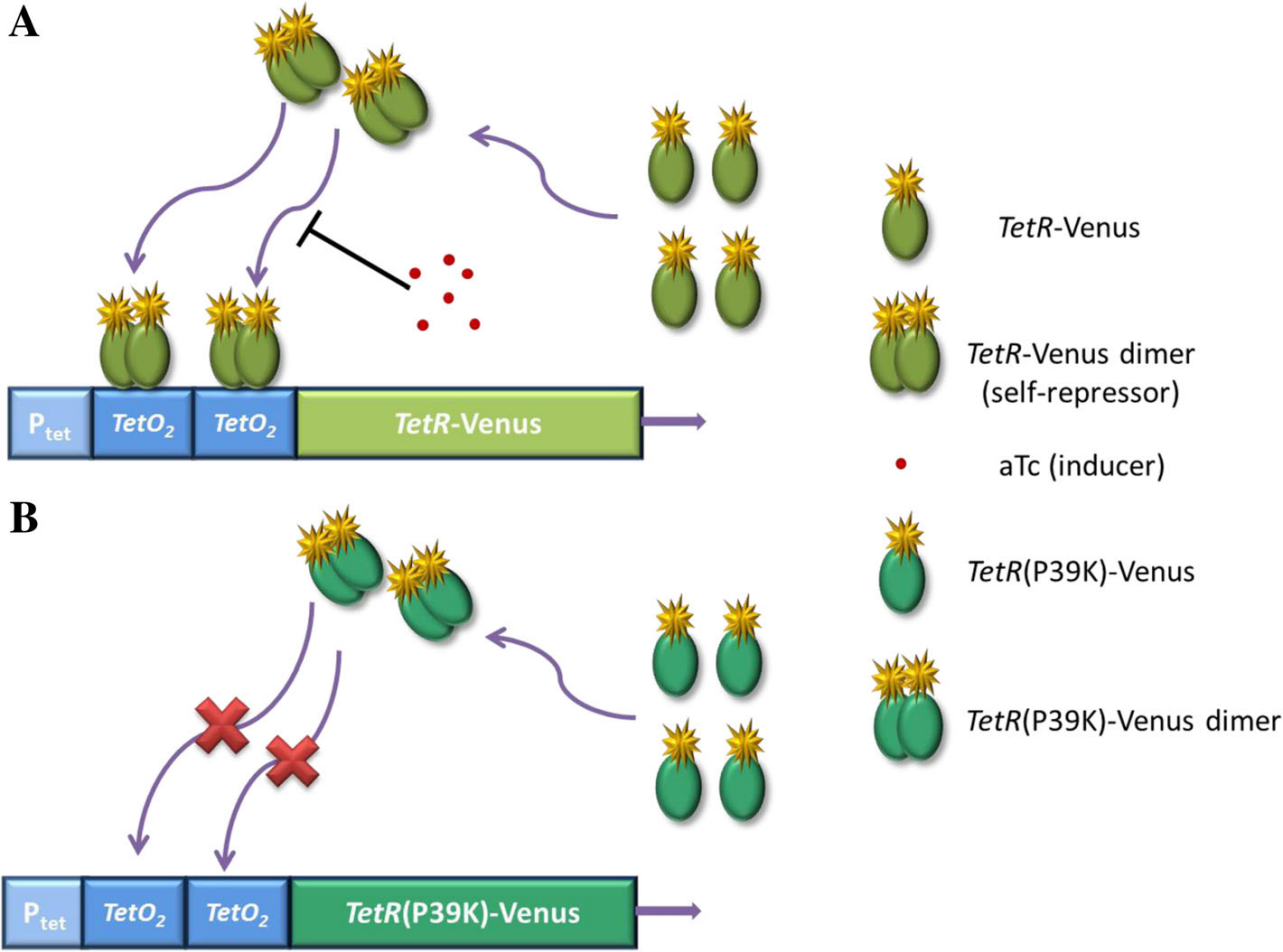
Schematic illustrations of the self-repressing gene circuit (MG::PR-8 T) and the non-self-repressing gene circuit (MG::PR-8 T-P39K). **a** Two *tet* operator sequences (T*etO*_2_) inserted downstream of the P*tet* promoter are bound by *TetR* self-repressor dimers. In the absence of aTc (the inducers), *TetR*-Venus dimers bind to the operators. This interaction prevents the binding of RNA polymerase, thereby inhibiting the *TetR*-Venus fusion protein synthesis. When aTc diffuse into the cell, they bind to *TetR*, inducing an allosteric conformational change in the repressor protein which releases it from DNA, allowing for the possibility of the gene being switched into the “on” state. All of these constitute a self-repressing gene circuit. **b** The *TetR*-P39K mutant is not capable of recognizing the operators and is unable to repress the *TetR*-Venus expression, constituting a non-self-repression gene circuit

In this study, we have designed and constructed a purely negative auto-regulation feedback loop circuit (self-repressing gene circuit) in *Escherichia coli* (*E. coli*). The Ptet promoter including two *TetO* operons controls the production of its repressor, *TetR*. Meanwhile, the *TetR* was fused with a fluorescence protein (Venus) for experimental measurements of the *TetR* expressions. The inducer, aTc (anhydrotetracycline), was introduced to regulate expressions of the self-repression system. In the presence of an inducer, the repressor *TetR* can change its conformation and dissociate from specific binding sequences of the DNA (*TetO*). This allows for the transcription of *TetR*-Venus (Fig. 1a). In order to avoid fluctuations in copy numbers of the plasmids, the constructed circuit in the plasmid was integrated into the chromosome of *E. coli*. We also constructed a series of self-repressing circuits with different affinities to the *TetR* protein (MG::PR-WT, MG::PR-1G) (Additional file 1: Figure S2). We chose MG::PR-8 T as the main circuit of this study for its stability and bimodal behavior. To compare this with our self-repressing circuit construction MG::PR-8 T, we designed a non-regulatory circuit as a control group: the MG::PR-8 T-P39K circuit (Fig. 1b).

### The expression distributions of the self-repressor gene circuit under microscopy

**Fig. 2:**
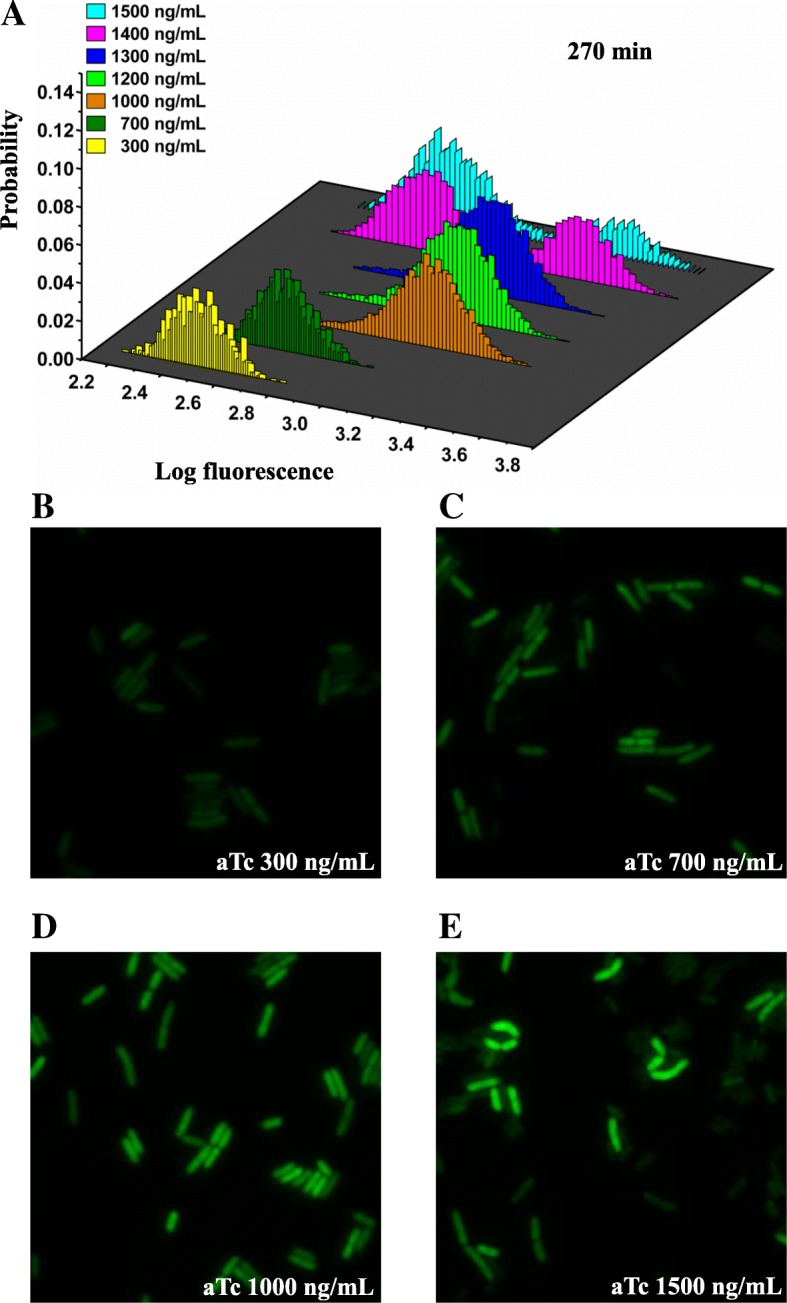
Experimental expression distributions of the self-repressing gene circuit (MG::PR-8 T) at different aTc concentrations observed under a microscope. **a** In M9 media with the inducer concentrations ranging from 300 to 1500 ng/mL of aTc, the resulting steady state fluorescence distributions show that the ratio of the populations of the bimodal fluorescence distributions depend on the aTc concentration. Seven color histograms represent different inducer concentrations. **b**–**e** Four representative fluorescence images at different concentrations of aTc (300, 700, 1000, and 1500 ng/mL) are selected

To obtain the expressions of *TetR* under different induction conditions, we measured the average fluorescence signals of the reporter protein Venus for the strain of MG::PR-8 T at different inducer concentrations (300–1500 ng/mL) across cell populations using a wide-field fluorescence microscope. Cells were collected and measured after being cultured in M9 medium and induced by aTc for 4~6 h to a logarithmic phase. To ensure accuracy of the expression distribution, we collected no less than 10^3^ cells to measure for each sample. All expression distributions under different induction concentrations are shown in Fig. 2. The results indicate that *TetR* expression distributions vary with inducer (aTc) concentrations. Under low inducer concentrations, the expression levels of the negative regulated gene circuit were quite low, and this gene can be considered to be in the “off” state for a long time. With increased inducer concentrations, the expression levels were significantly enhanced (Fig. 2a). From the results shown in the microscope, we can clearly see that when inducers are added to the system, the repressor *TetR* can no longer prevent the transcription of *TetR*. When the inducer concentrations are high enough (such as 1400 ng/mL and 1500 ng/mL), the steady state expression distribution can become bimodal, with two states of low and high expression levels. Meanwhile, the percentage of the cells in the low expression state gradually increases with the increase of the inducer concentrations (Fig. 2a). Under high inducer concentrations, the coexistence of both phenotypes characterized by the bimodal steady state distributions of the fluorescence intensities can be clearly seen (Fig. 2e). When we further compare the images in Fig. 2d and e, it is clear to see that one section of the cells in Fig. 2e is brighter, while other sections were dimmer, compared to most of the cells in Fig. 2d. As can be seen from the microscopy images, the morphologies of the bacteria cells are not influenced by the aTc inducers at a concentration level of 1500 ng/mL (Fig. 2e). The corresponding distributions of those images are given in Fig. 2a. It is important to note that cells grow normally (cell cycle 40–60 min) and have normal cell morphology even at high concentration of aTc (Fig. 4b, Additional file 1: Figure S8). Therefore, at this high aTc concentration, there seems no significant cytotoxicity on cells. In our control experiments, two state behaviors are not found in the strain of MG::PR-8 T-P39K with non-self-repressing gene circuit under the same conditions (Additional file 1: Figure S3, Additional file 1: Figure S7). This indicates that the two expression states of *TetR* were due to the self-repressing circuit, rather than other factors such as the influences of the inducers on the cell growth and ribosomal effects. Meanwhile, the other two strains of MG::PR-WT and MG::PR-1G even with self-repressing gene circuit displayed only single peak distributions (Additional file 1: Figure S3). These results suggested that the two states appear only under certain specific conditions. This might explain why previous studies did not see bimodality on negative auto-regulation system.

### Fano factor and inhibition curve

**Fig. 3:**
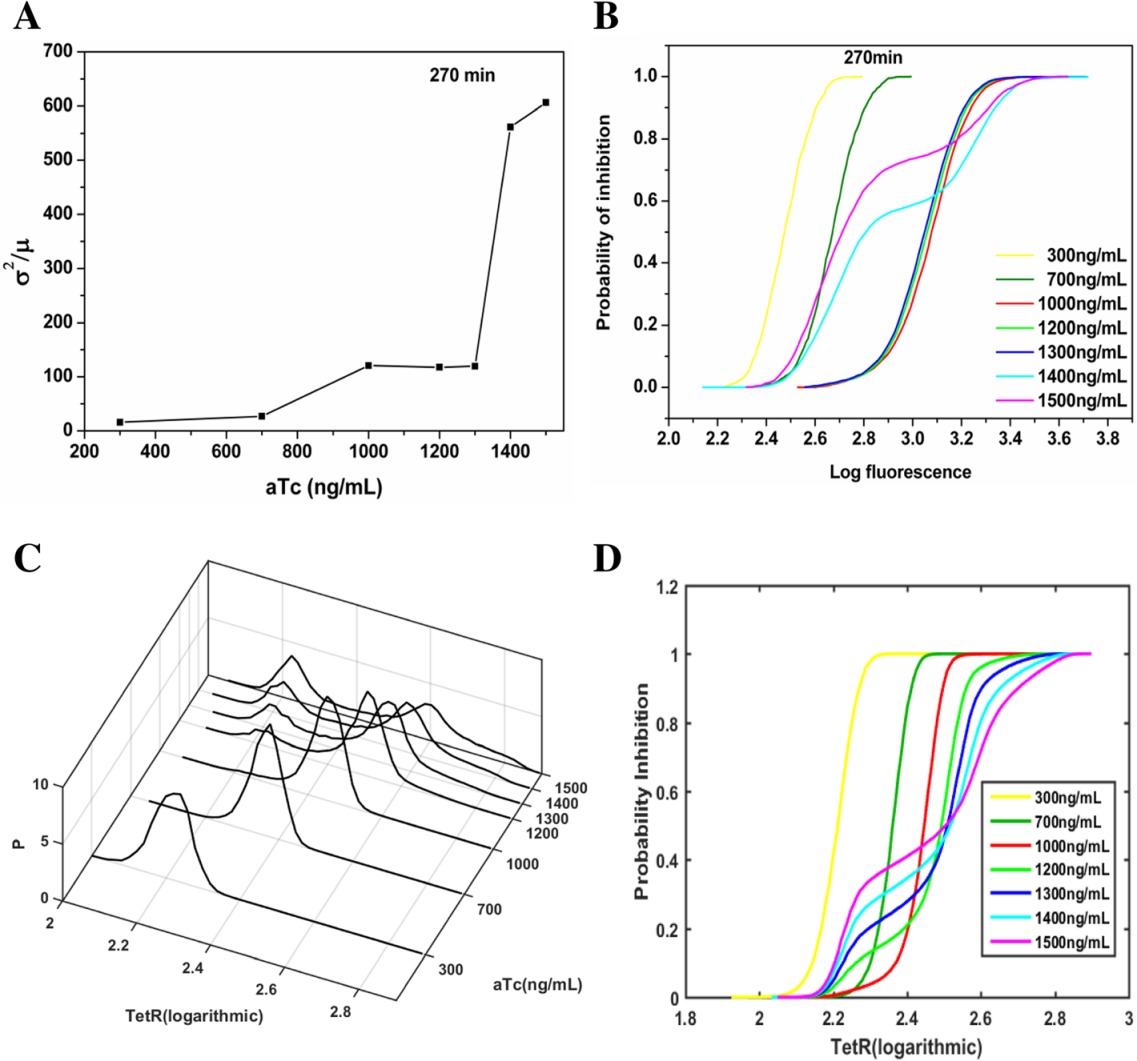
The Fano factor curves and the probability of inhibition curves of the self-repressing gene circuit. **a** Dose-response of the Fano factor (*F = σ*^*2*^*/μ*) of the *TetR*-Venus expression for the self-repressing gene circuit (MG::PR-8 T) at different inducer concentrations. The Fano factor is defined as *σ*^*2*^*/μ*, where *σ*^*2*^ and *μ* are the variance and the mean of the probability distribution. **b** The probability of inhibition curves of the MG::PR-8 T circuit at different inducer concentrations. Seven color histograms represent different inducer concentrations. The inhibition curves were obtained by the ratio of the cells with a fluorescence intensity lower than a certain value to the number of the total samples. **c** The probability distribution of the *TetR* proteins for the circuit of MG::PR-8 T with different concentrations of inducers from the stochastic simulation model. P(*n*) (*z* axis) represents the probability distribution of the *TetR* protein numbers (*x* axis), *n* at different numbers of inducer (aTc) molecules (y axis). **d** The probability of inhibition curves of the model. The cumulative distribution functions of the simulation were obtained from Fig. 3c. Seven color histograms represent different inducer concentrations

To further understand our experimental observations, we need to quantify the degrees of fluctuations. This can be measured by the Fano factor quantified as the variance of the observable (molecular number in the cell) divided by the mean value [35]. The Fano factor is equal to one (*F* = 1) if the distribution of the observable (the molecular number) is exactly Poisson. A large Fano factor implies significant statistical fluctuations deviating from Poisson. As the concentration of inducers increases, the Fano factor increases (Fig. 3a). When the concentration of inducer reached 1300 ng/mL, the change of Fano factor is more significant. Qualitatively, the Poisson distribution should be a good approximation for the individual “on” and “off” states when the observed distribution of fluorescence intensity is bimodal, because each gene state can produce proteins almost independently of gene switching. However, the overall Fano factor for the combined probability distribution of “on” and “off” states is much larger than the case of the zero or smaller inducer concentration. This is because the system is close to a two-peak (non-Poisson) distribution with different means summed together, producing large statistical fluctuations deviating from the single Poisson distribution. This indicates that two Poisson processes added together will not lead to a Poisson distribution. In this study, we measured the Fano factor quantified as the variance of the fluorescence observable (proportional to the molecular number) divided by the mean value to quantify the relative degree of the variance or fluctuations. In other words, the phenotypic noise strength, defined as the quantity δ^2^/μ, is a measure of the spread of expression levels in a population. Here we point out that even when the underlying population obeys the Poisson distribution, the fluorescence observable which is proportional to the population size may not have a Fano factor equal to one. It is worth emphasizing here that we care more about the trend or the changes of the Fano factor upon inducer concentrations rather than the absolute values here. This can help us to understand the degree of the spread or fluctuations in protein expression distribution upon inducer concentrations. The analysis of the coefficient of variation (CV) in Additional file 1: Figure S6 also illustrates this same conclusion.

Furthermore, we investigated the inhibition curve, which describes the proportion of the bacteria with a fluorescence intensity lower than a certain value (Fig. 3b). We can see that the proportion of the gene in its inhibited state first decreases at low concentrations of inducer (up to aTc concentration at 1200 ng/mL) and then increases as the inducer concentration becomes higher. More inducers introduce more interactions with the *TetR* molecules. This leads to a reduction in the number of free *TetR* (Additional file 1: Figure S15), and also slows down the effective binding and unbinding of the *TetR* to the DNA (Details in Additional file 1: SI on page 10). The reduction of the binding rate will lead to the decrease of inhibition capability, and the decrease of unbinding rate will lead to the increase of inhibition capability. When the concentration of inducer aTc is low (higher free *TetR* concentration), the binding rate has a greater influence (dimer with square dependence on the free *TetR* concentration) on the overall inhibition capability than the unbinding rate (linear dependence on the free *TetR* concentration [43–52]). Therefore, at low inducer concentrations, when the inducer concentration increases (up to aTc concentration at 1200 ng/mL), the free *TetR* concentration decreases. This leads to the reduction of the binding rate. Since binding has square dependence on the free *TetR* concentration and unbinding has linear dependence on the free *TetR* concentration, the binding has more significant influence than unbinding on inhibition capability at relatively low aTc concentration, i.e., relatively high free *TetR* concentrations. Therefore, the reduction of the binding leads to the decrease of the inhibition probability at this inducer concentration range (up to aTc concentration at 1200 ng/mL).

At certain concentrations of inducer aTc (1200 ng/mL), the binding rate and unbinding rate have the same effect on the inhibition capability. If the concentration of the inducer further increases (at this concentration range of aTc 1200–1500 ng/mL), the unbinding rate will have a greater influence on the inhibition capability, that is, the inhibition capability will increase as the inducer concentration increases. This is because unbinding has linear dependence on the free *TetR* concentration and binding has square dependence on the free *TetR* concentration; the unbinding has more significant influence than binding on inhibition capability at relatively high inducer concentration, i.e., at relatively low free *TetR* concentrations. At extremely high aTc concentrations (beyond 1900 ng/mL), the toxicity from aTc as antibacterial agent to the cells becomes effective. It is therefore not feasible to observe the healthy cell expression distribution at this extremely high concentration of aTc.

### The dynamics of *TetR* expression in real time

**Fig. 4:**
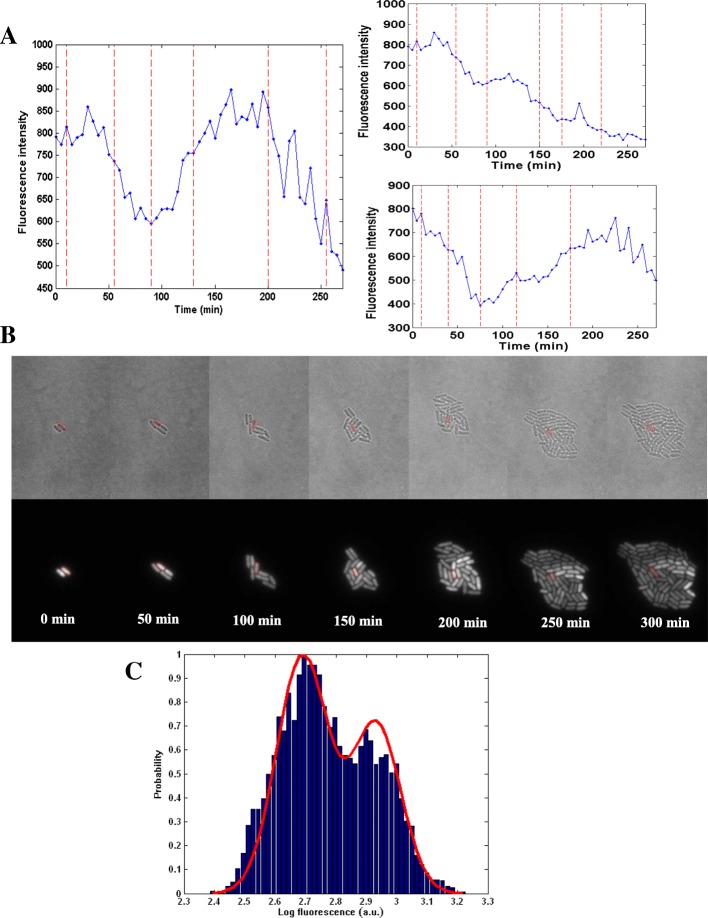
The mean fluorescence intensity distribution of the dynamical trajectories for MG::PR-8 T. Single-cell mean fluorescence intensities were captured every 5 min. 28 micro-colonies were tracked by time-lapse microscopy. **a** Three representative single cell fluorescence trajectories induced by 1500 ng/mL aTc. Points represent experimental fluorescence values. Red vertical dashed lines demarcate cell divisions. **b** The bright field and fluorescent field images of the corresponding measurements in the time-lapse experiment. The cells corresponding to the fluorescence trajectory in Fig. 4a are marked with red circles. The average of bacteria mean fluorescence intensity is 556, and the average cell cycle time is 46 min in this micro-colony. **c** The histogram gives the intensity distribution of the 163 single-cell fluorescence trajectories induced at 1500 ng/mL aTc collected from the time-lapse experiments. The red solid curve is the fitted intensity distribution from HMM

We have seen that the self-repressing circuit can give a bimodal distribution. In order to further explore the underlying mechanism of this behavior, we monitored the dynamics of *TetR* expression in real time. We tracked cells during their growth and division on a microscope with a FCS2 (Focht Chamber System 2, Bioptechs) system which provides aTc continuously to guarantee the cells growing in the right environments (continuous flow of adequate nutrients from fresh medium (M9) through the cells on agarose pad) and avoids potential issue of heterogeneity of the environments. As shown in Fig. 4b, upon aTc induction, two types of cell responses were observed: the fluorescence intensity either changed significantly or almost remained the same. When we track cells in real time, we can see that, some cells switch between bright and dim, while other cells stay with similar brightness (Fig. 4b). The resulting fluorescence distribution is thus bimodal and a fluorescence threshold can be defined for each cell in its most probable induction state. The use of a microfluidic device, coupled with cell tracking and fluorescence measurements, allows us to generate fluorescence trajectories for a single cell on reasonable time scales (~ 300 min) for a single trajectory. Based on this, we collected 28 micro-colony movies and chose 163 fluorescence trajectories. We observed that the trajectories of a single-cell fluorescence fluctuated significantly. We collected about 8200 fluorescence intensity data points corresponding to the selected trajectories. Several representative trajectories with significant fluctuations were shown to demonstrate the existence of two states (From Fig. 4a, b, Additional file 1: Figure S8, Additional file 2: Movie S1, Additional file 3: Movie S2 and Additional file 4: Movie S3).

### Two cell state identifications by hidden Markov chain modeling

In order to explore the underlying mechanism of the bimodality, we collected the statistics of the fluorescence intensity obtained from the trajectories. As expected, the distribution of these intensities exhibits two peaks, which is similar to the results in Fig. 2a. Here, it is important to note that, due to the different growth conditions of the cells between the steady state and time-lapse experiments, there should be certain differences on the expression distribution curve. However, the images taken under both steady state and time-lapse experiments show two group of cells (bright and dim cells) (Fig. 2e and Fig. 4b). This indicates that the two peaks should come from the two groups of the cells on both experiments. Therefore, we believe that the two state populations uncovered from the single-cell trajectories are the same two state populations uncovered in the steady state experiments. The emergence of the two peaks suggests that most of the initial cells are either in a high expression state or in a low expression state in their progeny. We then used a Hidden Markov Chain Model (HMM) [36] to fit the real-time trajectories and identify the cell states and then simulate the distribution of the fluorescence intensity (Fig. 4c). To assign protein expression states and the rates of inter-conversion between them, we performed data fitting using the HMM. From the HMM analysis, we obtained a correlation coefficient of 0.975 between the measured and simulated trajectories after identifying the cell states and quantifying their switching rates. The simulated distribution fits with the measured distribution well. From the HMM analysis, we further determined the center positions of the peaks to be at 2.690 and at 2.933 in logarithm of fluorescence intensity. The variances of the individual peak distributions are at 0.085 and at 0.080, respectively. For our system, the probability in the high expression state is around 0.401, and we can also see that the probability in the low expression state is around 0.599.

In the high expression state, the system will continue its behavior with a probability of 0.963 (the switching or residence time will be discussed in the next section). There is a small chance, with the probability of 0.037, to switch to the low expression state from the high expression state. Meanwhile, there is additionally a probability of 0.023 that the system will switch to the high expression state from the low expression state, instead of remaining in the low expression state.

### The average residence times of the protein expression states

To estimate the average residence times of the protein expression state, we distinguished the states from the trajectories using HMM analysis and calculated the residence times of each state (Additional file 1: Figure S11). For each trajectory, we counted the total residence times and the number of the state changes. The average residence times were calculated as the quotient of the total residence times and the number of states changed.

The length of the test fluorescence trajectory is finite and limited. This may lead to some errors in estimating the transition times. We take this into account in determining the time scale of the transitions (SI P8-P9). The average residence time of the high expression state is estimated to be about 92~103 min, and that of the low expression state is estimated to be about 151~182 min. The average residence time can be used to quantify the switching time between two cell fates. Therefore, the switching time from high (low) expression to low (high) expression can be estimated to be about 92~103 (151~182) min. Through fluctuations, the bimodal distribution can be maintained in a dynamic balance between the high expression “on” state and the low expression “off” state. When the inducer concentration is fixed, the increasing number of proteins will promote the inhibition probability of gene switching. Therefore, the cells in the high expression state will have a tendency to migrate to the low expression state. Conversely, the cells with low expressions will be more likely to move towards the “on” state. Therefore, the cells in the low expression state will also have a tendency to migrate to the high expression state.

### Physical origin of the two cell fates

Intuitively, from a molecular perspective, we know that the transcription process is suppressed when the promoter site of the DNA is occupied by a repressor (the gene is “off”) and enhanced when the repressor is dissociated from DNA (the gene is “on”).

According theoretical studies [32], when the binding/unbinding is much faster relative to the synthesis/degradation, the gene regulatory network is under adiabatic limit. In this case, the “on” and “off” of gene states switch rapidly, but the protein molecules synthesize or degrade much slower in this self-repressor circuit. Therefore, the expression of the protein and associated gene are strongly coupled together. As soon as the proteins are synthesized, they will immediately bind to the gene. Therefore, the protein is always repressed (self-repressor) and the protein expression displays a single peak at the long time scale. When the binding/unbinding is lower or comparable relative to the synthesis/degradation, the gene regulatory network is in non-adiabatic limit. In this case, the gene state changes slowly. After the proteins are synthesized, they will take certain amount of time for binding. During this period of time, the gene state can be on without binding while after this time the gene will be off due to the protein binding to the gene. Therefore, there is a fraction of time the gene is in on and off state. This leads to the emergence of the protein distribution with two distinct peaks showing two different gene states.

Although we did not directly measure the binding/unbinding rate of *TetR*, recent single-molecule experiments [43–52], using force spectroscopy, fluorescence microscopy, and analog computation have determined the unbinding rate of some proteins bound to the DNA. The results showed that the unbinding rate depends on the concentration of freely diffusing proteins in the solution. For the unbinding mechanism, Paramanathan et al. proposed a universal rapid rebinding model to quantitatively explain the kinetic process of competitor-induced dissociation. The protein bound to DNA undergoes rapid dissociation and rebinding in the vicinity of the inhibition site. Other proteins in this region can interact with the inhibition site to accelerate the unbinding rate. We suggest that this model can explain our experimental observed behaviors well. For the binding reaction, *TetR* dimer binds to DNA inhibition site to repress the gene expression, two *TetR* monomers combine to form a *TetR* dimer, and then the binding reaction can be simplified to a reaction between the gene and two *TetR* monomers. This leads to the quadratic dependence of the binding rate on the *TetR* concentrations. For the unbinding reaction, according to the rebinding model, the gene will react with the competitor. Due to the presence of the inducer, the conformation change of *TetR* will lead the *TetR* dimer to convert into monomers [53], so the competitor can be chosen as *TetR* monomer. This leads to the linear dependence of the unbinding rate with respect to the free *TetR* concentrations.

For dimer binding, the binding rate is expected to be proportional to the square of the concentration of the free *TetR*. The binding of aTc and *TetR* will lead to a decrease in the number of free *TetR* (Additional file 1: Figure S15). This will further lead to a decrease in the binding and unbinding rate between the *TetR* and the promoter. Therefore, when the inducer concentration is low, increasing the inducer concentration will decrease the free *TetR* and slow down the binding and unbinding rate of free *TetR* to the promoter. This will increase the probability of the gene being at the “on state” and “off state.” When the free *TetR* concentration is not too low, the effect of binding (quadratic dependence on the free *TetR* concentration) is more significant compared to the effect of unbinding (linear dependence on free *TetR* concentration). As the aTc increases, free *TetR* decreases. This reduces the binding rate more significantly than the unbinding rate. The less effective binding is expected to lead to more chances of genes being at on state. Furthermore, the binding and unbinding rates are usually faster than the degradation rate of *TetR*, the resulting synthesis is faster. As a result of all above, the higher expressions emerge. This explains the shift of the expression peak from low to high as inducer concentration increases.

When the inducer concentration further increases to sufficiently high values, the free *TetR* molecules will be fewer, the binding and unbinding rate will also be lower. This will increase the residence time of genes being at “on state” and “off state.” At the “on state,” more *TetR* will be generated, and then bind with the inducer, corresponding to the high expression peak. However, when the free *TetR* concentration is very low, the effect of unbinding (linear dependence on the free *TetR* concentration) is more significant compared to the effect of binding (quadratic dependence on free *TetR* concentration). As the aTc increases further, free *TetR* decreases. This reduces the unbinding rate more significantly than the binding rate. The less effective unbinding is expected to lead to more chances of genes being at off state. As a result, the lower expressions emerge. This explains the shift of the expression peak from high to low as inducer concentration further increases.

When the average residence time of “off state” is longer than the average degradation time of *TetR*, the free *TetR* and the *TetR* binding with aTc will be degraded significantly. The resulting synthesis and degradation of the proteins can reach the steady state. This will make the low expression peak more stable. Further increases of the inducer concentrations will lead to less free *TetR* and the longer residence time of “off state.” This leads to greater weighting of low expression peak rather than high expression peak.

### Stochastic simulations of bimodality

To verify our explanation about emergence of two states on our *TetR* self-repressor system discussed above, we further explored the stochastic dynamics of self-regulative feedback genes through a mathematical model, which can be used to explain and simulate the experimental observations (Fig. 3c). The mathematical model clarifies the underlying mechanism of how bimodality emerges. Under faster regulation binding, the self-repressor is forced to stay in the repressed state. This is because once produced, the regulatory protein immediately binds to the gene and therefore represses protein production. In our study, slower binding of the regulatory protein to the gene is realized through the inducer binding to the regulatory protein, which effectively blocks the ability of the protein to bind to the promoter. Under slower regulatory binding, the self-repressor may function in two different ways: it may bind to the DNA for some time and repress protein production, or unbind from the DNA for some time, leading to increased protein production. This generates two cell phenotypes. Furthermore, due to the intrinsic statistical fluctuations of the number of proteins, there is a possibility of switching between the high expression and low expression state. We have observed such phenotypic switching in real-time experiments. The simulation results are consistent with the experimental observations for the emergence of the two peak distributions in Fig. 2a.

On the other hand, the trajectories in Fig. 4a and Additional file 1: Figure S8 showed comparable growth rates in high expression state and in low expression state. It is possible that high expression cells in our study have not reached the threshold for significant metabolic burden to slow down the growth. The inhibition curves of the different inducer concentrations in Fig. 3b and the dynamic balance by intrinsic fluctuations also imply that the bimodality of the protein expression distribution is not due to cell growth. The simulation results of Fig. 3d are also consistent with the experimental finding for inhibition probability shown in the Fig. 3b.

### Biomodality in self-repressing circuits at different affinities

We constructed three self-repressing circuits with different affinities to the *TetR* protein [38] (relative affinity of *TetR* between WT, 8 T, and 1G is 100, 79, and 13). We chose MG::PR-8 T as the main circuit of this study for its stability and bimodal behavior since we did not observe good reproducible bimodal behavior on the other two circuits under our current experimental conditions. We hypothesize that the differences in affinities between the gene circuits and the *TetR* protein are the main cause of the differences in behaviors. It is reasonable to expect that the same behavior such as bimodal behavior for the systems with different affinities requires different experimental conditions.

Here, we can give a more detailed possible explanation from the simulations. In the simulation, we used rate parameter f/h to quantify and clarify the important role of affinity in the self-repressor. Different affinities correspond to different equilibrium constants f/h in the inverse fashion. We started the unbinding/binding ration f/h corresponding to 8 T circuit. Since the relative affinity of 1G circuit is lower than 8 T, to stimulate the affinity of 1G circuit, we increased the value of f/h, and the results were given in Additional file 1: Figure S16. Bistability can still be observed but at much higher aTc concentration. As mentioned before, the cells can hardly survive in such an environment. This may explain why we did not observe bimodal behavior in the strain of MG::PR-1G. On the other hand, we also stimulated the WT circuit with higher affinity. The results are shown when the unbinding/binding ratio of f/h decreases, in Additional file 1: Figure S17. Although bimodal behavior in principle can be reached at lower aTc concentrations, it is very difficult to distinguish and clearly separate the two states due to the fluctuations, noises and errors at lower concentrations of proteins in the strains of MG::PR-WT. In this study, the behavior of MG::PR-8 T is sufficient to illustrate that the self-repressing circuits can become bimodal. Therefore, we conclude that the bimodality can emerge for self-repressor systems under certain experimental conditions.

### Biomodality and trend of biomodality upon inducer impacts and their relationships to *TetR* concentration dependence of binding/unbinding regulations

Another issue of concern is that whether *TetR* concentration dependence is necessary for the bistability to occur. Based on the simulation results, we can see that the distribution with the unbinding rate independent on the concentration can still be bimodal. In other words, when the concentration dependence for unbinding is removed, we can also reach the bimodal distribution, as shown in the Additional file 1: Figure S18. However, the trends and shapes of the expression distribution with respect to the inducer concentration are not consistent with the experimental observations. We can see that when the inducer concentrations are high enough (such as 1400 ng/mL and 1500 ng/mL), the steady state expression distribution can become bimodal, with two states of low and high expression levels, respectively. Meanwhile, the percentage of the cells in the low expression state gradually increases with the increase of the inducer concentrations (Fig. 2a). When the concentration dependence of unbinding is removed, the percentage of the high expression state gradually increases with the increase of the inducer concentrations when the bimodality is emerged, which is not consistent with the experimental observation.

To illustrate this more specifically, let us consider the following scenario. When the concentration of inducer is low, the concentration of free *TetR* is high, and the gene is mostly in the off state. Then, the gene expression would have a single peak distribution. Increasing the inducer concentration will decrease the free *TetR* concentration and slow down the binding rate of free *TetR* to the promoter, this will increase the probability of the gene being at the “on state.” If we assume that the unbinding rate is constant, the probability of the gene being at the “off state” is invariant. Hence, the shift of gene expression peak is from low to high.

When the inducer concentration is further increased, the probability of “on state” would continue to increase. Due to the very fast expression rate, we will reach a higher expression peak corresponding to the higher probability of “on state.” As the inducer aTc concentration increases further, the free *TetR* decreases and the probability of “on state” continues to increase, this leads to greater increase of the weight of high expression peak rather than low expression peak (Additional file 1: Figure S18). In this condition, the simulation results with unbinding rate independent of TetR concentration are not consistent with the experimental observations.

## Discussion

Our study shows explicitly in this concrete gene circuit that different cell fates can emerge not only from the changes in the genes (such as mutations) but also from the changes in regulatory wirings or links through microenvironments without altering the gene itself. In fact, even when the topology of the wiring for the underlying gene regulatory network is fixed, there is still a possibility of cell phenotypic changes due to the changes in the regulation strengths induced by the environment. Furthermore, we observed in both real-time experiments and simulations that the cell phenotypes or fates can be switched from one to the other. We also obtained the average time of this switching which quantifies how difficult it is to communicate globally from one cell fate to the other. Therefore, using real-time trajectories, we determine both the speed and the underlying processes of the cell fate decision-making/phenotypic state-switching.

Epigenetic effects are often challenging to study in eukaryotic cells. Our study in bacteria illustrates how the environments can influence the cell fates and cell fate decision-making in a controllable way. The experiments in bacteria are relatively easy and straightforward to perform and control. The epigenetic and micro-environmental effects can be mimicked through the modulation of inducers in our study. The essence of the effects of the epigenetics is the change of the effective time scales of the regulatory binding/unbinding. Here the introduction of the inducers into the culture medium changes the microenvironment of the cell and leads to the changes of the time scales of the regulatory binding/unbinding, mimicking the effects of the epigenetics. This is an advantage of our approach. We plan to apply our method to a variety of core regulatory motifs and modules in the gene networks to investigate how the microenvironments or epigenetics influence the cell fates and the cell fate decision-making processes.

## Conclusions

From the results of this experimental study, we suggest that different phenotypes can emerge from the same genotype under environmental changes, in this case that introduction of an inducer to a self-repressor circuit can, in certain conditions, lead to the emergence of two cell fates rather than the expected one fate. We observe the switching dynamics for cell fate decision-making between these two types of populations and propose a model to show that slow binding/unbinding, rather than stochastic fluctuations, may underlie the emergence of bimodality.

## Materials and methods

### Strains and plasmid construction

The tetracycline inducible promoter (P*LtetO-1*) and a strong ribosome binding site (RBS, BBa_B0030) were obtained from the plasmid of pZE11, which was kindly provided by Bujard [26]. The P*LtetO-1* promoter has two *TetO2* operator sites. This promoter is tightly repressed by the Tn10-encoded Tet repressor (*TetR*) and can be activated by a supply of anhydrotetracycline (aTc).

All strains were constructed using standard cloning techniques. The sequences of the PCR primers used in this study are shown in Additional file 1: Table S1. The fusion PCR was used to construct the *TetR*-Venus fusion protein. The protocol is based on two rounds of PCR: The first PCR uses *TetR*-F/Linker-R and Linker-F/Venus-R as primer pairs, whose templates are the plasmid pBR322 (Invitrogen™) and *E. coli* SX4 genomic DNA from Xie [23], respectively. In the second PCR, the fragments from the first round were amplified and fused using primers *TetR*-F/Venus-R, resulting in the *TetR*-Venus fragment. The *TetR*-Venus fusion protein was connected with the flexible linker, which provided the proper distance between *TetR* and Venus to avoid interference.

The entire *TetR*-Venus fragment was digested with *Kpn*I and *Xba*I and ligated into the pZE11 vector backbone, which placed the *TetR*-Venus under the control of the P*LtetO-1* promoter, yielding the plasmid pZE11-P*LtetO-1*-*TetR*-Venus.

### Recombination of chromosomes

*E. coli* K-12 MG1655 was chosen as the host strain of the self-repressing circuit model system due to its hypotoxicity and its known genomic sequence. It demonstrates good performance for the foreign protein expression and has been used in genetic studies in laboratories worldwide. This is crucial for the expressions of the regulatory proteins in the self-repressing circuit.

The CRIM (conditional-replication, integration, and modular) plasmid pAH150, the corresponding helper plasmids pINT-ts, and *E. coli* MG1655 (F-λ-*ilvG*-*rfb*-50*rph*-1), were kindly provided by Wanner [37]. The vectors are designed to integrate into the bacterial chromosome at different phage attachment sites. The P*LtetO-1-TetR*-Venus was inserted into the pAH150 at the *Xho*I and *Xba*I sites, and as-prepared plasmid pAH150-P*LtetO-1-TetR*-Venus (Additional file 1: Figure S1) was transformed into the DH5α λ pir^+^ strain for amplification. The resultant plasmids were integrated into the chromosome of the MG 1655/pINT-ts strain by standard electro-transformation protocol. A single copy of the integrant was screened from the transformants: the self-repressing gene circuit MG1655::P*LtetO-1-TetR*-Venus (MG::PR-WT).

### The construction of other self-repressing gene circuits

We mutated the operators to change their affinities to the repressor (Additional file 1: Figure S2). The mutated P*LtetO-1* gene circuits were amplified by PCR using pAH150-P*LtetO-1-TetR*-Venus as a template, Ptet-X-F (*X* = 8 T, 1G) as a forward primer and Venus-R as a reverse primer. These were then inserted into the pAH150 plasmid [38]. The constructed plasmids were integrated into the chromosome of MG 1655 to obtain MG::PR-8 T. We thus constructed a series of self-repressing circuits with different affinities to the *TetR* protein.

### Construction of the control non-regulatory gene circuit

To investigate whether the bimodality phenomena is produced by the self-repressing circuit, we designed a non-regulatory gene circuit as a control (Fig. 1b). The *TetR* mutant was introduced by site-directed mutagenesis with the pAH150-P*LtetO-1*-8 T-*TetR*-Venus plasmid as a template and P39K-F and P39K-R as a forward and a reverse primer according to standard procedures. The purified product was integrated into MG1655 as mentioned above, generating the MG1655::P*LtetO-1*-8 T-*TetR*-P39K-Venus (MG::PR-8 T-P39K) circuit. The differences in binding affinities of the mutated *TetR* (*TetR*-P39K) and the wild-type *TetR* (wt*TetR*) may be attributed to their different interactions with the Tet operator. The mutant amino acid decreased the interaction strengths between *TetR*-P39K and *TetO,* without affecting *TetR* and aTc interactions [39]*.*

### Microscopy measurements and image analysis

Cells were cultured overnight in M9 minimal media supplemented with vitamins (MEM vitamin solution, Gibco) containing appropriate antibiotics by shaking at 250 rpm at 37 °C. For microscopy measurements, the overnight cultures were diluted 1000-fold into 5 mL of M9 media with different aTc concentrations (300–1500 ng/mL) (Additional file 1: Table S2) and were continuously shaken at 250 rpm. After four and a half hours, the cultures were washed twice with sterile water by centrifugation. The resulting pellet was re-suspended in M9 media with appropriate antibiotics to an OD_600_ of 0.2–0.4. Small gel pads were prepared using 3% low-melting-temperature agarose in M9 media between microscope slides. 0.4 μL of the prepared cell cultures were dropped on each pad for imaging using a fluorescent microscope.

The fluorescence values of the single cells were measured using an inverted fluorescence microscope (Ti-E, Nikon) with automated stage and focus, equipped with a × 100 oil immersion objective. We applied 514 nm of an argon ion laser and set the output power at 5 mW (only 10% of the laser beam into the microscope objective). For each experiment, images of about 3000 cells on each slide were collected using a cooled EM-CCD camera (iXon3EM DU-897, Andor, Connecticut, USA). These images were acquired by Metamorph. Data analysis was accomplished through a combination of manual and automated analysis using custom MATLAB code (Schnitzcells).

### The time-lapse experiment and image analysis

Overnight cultures in M9 media at 37 °C were diluted 1000-fold into M9 media with 1500 ng/mL of aTc and agitated at 37 °C for 3 h. According to the preliminary experiment, the concentration of aTc in the time-lapse experiment was set as 1500 ng/mL. 0.4 μL of washed and re-suspended cell cultures in M9 media were dropped onto an agarose pad (3% agarose in M9 media with 1500 ng/mL of aTc). The agarose pad was covered with a cover slide, resulting in cell placement into a FCS2 chamber [40]. Bioptechs-FCS2 system settings were applied: we used a media flow rate of 1-2 μL/s, a media temperature of 37 °C, and a microscope objective temperature of 35 °C. All settings were adapted for optimizing the *E. coli* cell monolayer growth without physically disturbing the cells. The laser can be tuned at 514 nm for the Venus protein, and the output laser power was set at 3 mW (only 10% of the laser beam up into the microscope objective). The fluorescent images were periodically captured and recorded in the transmitted and fluorescent channels every 5 min over a period of around 6–7 h with an exposure time of 50 ms. All images were acquired using both bright field imaging and fluorescent field imaging.

Based on the custom analysis codes (Schnitzcells) compiled by MATLAB from Elowitz’s lab [41, 42], we applied some modifications to adapt to our requirements. Such improved analysis codes were used to identify and follow the cells, to reconstruct their lineage in time-lapse analysis by fluorescence imaging, and to identify cells in statistical analysis. Finally, we collected the fluorescent data of each cell at each time point for the following discussion.

### A simple mathematical model explaining the self-repressor bimodal distribution

We simulated the stochastic dynamical process of the self-repression gene circuit under intrinsic fluctuations from the finite number of molecules in the cell. The dimer consists of two regulator proteins *TetR* where each can be bound on to the DNA promoter with rate ½ h n (n-1). Meanwhile, the dimer can be dissociated from the promoter with rates f *n, and this rate is the unbinding rate which is proportional to the number (*n*) of free *TetR*. [50]. The binding and unbinding reactions of TetR with DNA are listed as follows:

$$ {\displaystyle \begin{array}{l}{A}^{11}+2 TetR\overset{h}{\to }{A}^{10},\kern0.5em {A}^{10}+ TetR\overset{f}{\to }{A}^{11}+2 TetR+ TetR\\ {}{A}^{11}+2 TetR\overset{h}{\to }{A}^{01},\kern0.5em {A}^{01}+ TetR\overset{f}{\to }{A}^{11}+2 TetR+ TetR\\ {}{A}^{10}+2 TetR\overset{h}{\to }{A}^{00},\kern0.5em {A}^{00}+ TetR\overset{f}{\to }{A}^{10}+2 TetR+ TetR\\ {}{A}^{01}+2 TetR\overset{h}{\to }{A}^{00},\kern0.5em {A}^{00}+ TetR\overset{f}{\to }{A}^{01}+2 TetR+ TetR\end{array}} $$

where *A*^ij^ denotes the unbound (bound) state of the gene that synthesize the *TetR* protein (*i* represents one regulator protein of the dimer binding and *j* represents another regulator protein of the dimer binding, 1 and 0 represent on and off state of the genes, respectively). For the self-repression gene circuit, *A*^11^ denotes that the gene is completely switched “on” when the operator of the gene is unoccupied and active. *A*^00^ denotes that the gene is completely switched “off” when the operator of the gene is occupied and repressed. For the binding reaction, *TetR* dimer binds to DNA inhibition site to repress the gene expression, two *TetR* monomers bind to form a *TetR* dimer, and then the binding reaction can be simplified into a reaction between the gene and two *TetR* monomers. This leads to the quadratic dependence of the binding rate on the *TetR* concentrations. For the unbinding reaction, according to the rebinding model, the gene can react with the competitor. Due to the presence of the inducer, the conformation change of *TetR* can lead to the monomerization of *TetR* dimer [53], so the *TetR* monomer can act as competitor. This leads to the linear dependence of the unbinding rate with respect to the free *TetR* concentrations.

We combined the transcription and translation steps for simplicity. Then, the transcription-translation step is described as follows:

$$ \varnothing \overset{g_{ij}}{\to } TetR\ \mathrm{for}\ {A}^{ij},\kern1.25em TetR\overset{k_1}{\to}\varnothing $$

where Ø is used to represent a protein sink or source. The source for synthesis comes from the gene activations while the sink comes from the protein degradations. *g*_ij_ is the protein synthesis rate when promoters are active or inactive for gene state *A*^ij^, respectively. When we added the inducers into the cells, the inducers aTc were found to bind with the regulator protein *TetR*. The resulting binding complex cannot bind effectively to the promoter and therefore cannot have a significant influence on regulating gene transcription for synthesizing the protein. The binding complex of aTc and *TetR* may also degrade. This process can be described as follows:

$$ TetR+{I}_{aTc}\underset{a}{\overset{b}{\rightleftarrows }} Ta,\kern1em Ta\overset{k_2}{\to}\varnothing $$

$$ {E}_{aTc}\underset{c}{\overset{c}{\rightleftarrows }}{I}_{aTc} $$

where *Ta* denotes the binding complex of *TetR* and aTc. These binding complexes of aTc and *TetR* cannot occupy the binding site of the promoter. *I*_aTc_ and *E*_aTc_ denote the internal and the external inducer (in and out of the cells). The internal and external inducers can mutually diffuse through the cell membrane with diffusion coefficient *c*. We define *ω* = f/k, which quantifies the ratio between the unbinding rate of *TetR* to the promoter and the speed of the *TetR* protein degradation. Likewise, the equilibrium constant *X*eq = f/h quantifies the relative balance between dissociation and binding of *TetR* to the promoter (Additional file 1: Table S3) and is directly related to the affinity.

## Additional files

Additional file 1: Supplementary Text. **Table S1–S3. Figure S1–S18.** **Table S1.** PCR primers. **Table S2.** The corresponding relationship between the mass concentration and the molar concentration. **Table S3.** Rate constants in mathematical model. **Figure S1.** The recombinant plasmid map. **Figure S2.** Diagram of synthetic circuit constructs. **Figure S3.** The distributions of mean fluorescence intensity of MG::PR-WT and MG::PR-1G. **Figure S4.** The distributions of mean fluorescence intensity of MG::PR-8 T. **Figure S5.** Total probability of the DNA in the bound state. **Figure S6.** Fano factor curves and the overall coefficient of variation (CV) curves. **Figure S7. ** Expression distributions of MG::PR-8 T-P39K. **Figure S8.** The representative trajectories for the MG::PR-8 T strain. **Figure S9.** The fluorescence images of MG::PR-8 T-P39K. **Figure S10.** A representative trajectory collected at 50 ng/mL. **Figure S11.** The schematic diagram of calculating the average residence time (each frame lasts 5 min). **Figure S12.** The bifurcation diagram in total number of *TetR* (in nmol/L) molecules and aTc (in ng/mL) molecules. **Figure S13.** The bifurcation diagram in total number of *TetR* (in nmol/L) molecules and aTc (in nmol/L) molecules. **Figure S14.** The simulated steady state probability distribution in total *TetR* (in mol/L) and aTc (in mol/L) molecules. **Figure S15.** The simulated steady state probability distribution in free *TetR* (in mol/L) and aTc (in mol/L) molecules. **Figure S16.** The simulated steady state probability distribution with much higher aTc concentration. **Figure S17.** The simulated steady state probability distribution in different aTc concentrations. **Figure S18.** The simulated steady state probability distribution in different aTc concentrations when the unbinding rate is assumed not to be concentration dependent. (DOCX 4940 kb)

Additional file 2: **Movie S1.** This movie file shows a time-lapse microscopy of MG::PR-8 T strains continuously induced with 1500 ng/mL of aTc at a constant temperature of 37 °C. The total time of the movie is 225 min with a rate of one image every 5 min. (AVI 203 kb)

Additional file 3: **Movie S2.** This movie file shows a time-lapse microscopy of MG::PR-8 T strains continuously induced with 1500 ng/mL of aTc at a constant temperature of 37 °C. The total time of the movie is 375 min with a rate of one image every 5 min. (AVI 273 kb)

Additional file 4: **Movie S3.** This movie file shows a time-lapse microscopy of MG::PR-8 T strains continuously induced with 50 ng/mL of aTc at a constant temperature of 37 °C. The total time of the movie is 112 min with a rate of one image every 8 min. (AVI 74 kb)

## Data Availability

All data generated or analyzed during this study are included in this published article and its supplementary information files.
