# Supplementary material for: The emergence of the two cell fates and their associated switching for a negative auto-regulating gene
Source: BMC Biol. 2019 Jun 15;17:49. doi: 10.1186/s12915-019-0666-0 (PMC6570905; doi:10.1186/s12915-019-0666-0)
Supplement: Supplementary file 1 — Supplementary Text. Table S1–S3. Figure S1–S18. Table S1. PCR primers. Table S2. The corresponding relationship between the mass concentration and the molar concentration. Table S3. Rate constants in mathematical model. Figure S1. The recombinant plasmid map. Figure S2. Diagram of synthetic circuit constructs. Figure S3. The distributions of mean fluorescence intensity of MG::PR-WT and MG::PR-1G. Figure S4. The distributions of mean fluorescence intensity of MG::PR-8 T. Figure S5. Total probability of the DNA in the bound state. Figure S6. Fano factor curves and the overall coefficient of variation (CV) curves. Figure S7. Expression distributions of MG::PR-8 T-P39K. Figure S8. The representative trajectories for the MG::PR-8 T strain. Figure S9. The fluorescence images of MG::PR-8 T-P39K. Figure S10. A representative trajectory collected at 50 ng/mL. Figure S11. The schematic diagram of calculating the average residence time (each frame lasts 5 min). Figure S12. The bifurcation diagram in total number of TetR (in nmol/L) molecules and aTc (in ng/mL) molecules. Figure S13. The bifurcation diagram in total number of TetR (in nmol/L) molecules and aTc (in nmol/L) molecules. Figure S14. The simulated steady state probability distribution in total TetR (in mol/L) and aTc (in mol/L) molecules. Figure S15. The simulated steady state probability distribution in free TetR (in mol/L) and aTc (in mol/L) molecules. Figure S16. The simulated steady state probability distribution with much higher aTc concentration. Figure S17. The simulated steady state probability distribution in different aTc concentrations. Figure S18. The simulated steady state probability distribution in different aTc concentrations when the unbinding rate is assumed not to be concentration dependent. (DOCX 4940 kb) [file 12915_2019_666_MOESM1_ESM.docx]

**Additional file 1**

**Supplementary Text**

**Self-Repressing Gene Circuit and Non-Regulatory Gene Circuit**

In living cells, gene expression is regulated by complicated genetic networks, which are often made of smaller gene modules (27, 28, 32, 33, 54-58). Thus it is important to understand these motifs, as they are the basic building blocks of complex networks (31-33, 57-60)*.* In this study, we have designed and constructed a simple self-repressing gene circuit in *E. coli* through a pure negative auto-regulation feedback loop (Figure 1A). When the inducers were not introduced, the expressed *TetR* was shown to strongly repress the expression of *TetR*-Venus through its binding to T*etO_2_* operator sites. However, after binding two inducer molecules, each *TetR* dimer underwent a conformational change, which prevented the free dimers from binding to the operator sites. As a result, *TetR*-Venus continued to be expressed (Fig. 1 and Additional File 1: Fig. S2). In order to reduce the impact of the fluctuations in the copy numbers of plasmid, the constructed circuit in the plasmid was integrated into the chromosome of *E. coli*. We chose MG1655::P*LtetO-1*-8T-*TetR*-Venus (MG::PR-8T) as the main circuit of this study (Additional File 1: Fig. S2). In order to compare this with MG::PR-8T, we designed a non-regulatory gene circuit as a control group: the MG::PR-8T-P39K circuit (Fig. 1B) (61). In order to facilitate observation and quantitative analysis, *TetR* of the transposon Tn10 was fused to the Venus, a variant of yellow fluorescent protein (YFP), and was placed downstream of the P*LtetO-1* promoter containing two *TetO_2_*. The negative feedback circuit was then integrated into the chromosome. The fluorescent measurements were carried out with a FCS2 platform tailored for fluorescence microscopy.

**Expression Distributions of the Self-Repressor Gene Circuit in Microscopy**

Venus fluorescence intensities of the MG::PR-8T circuit were monitored at various aTc concentrations from 300 to 1500 ng/mL as shown in Additional File 1: Figure S4. The Venus fluorescence expression distribution of the MG::PR-8T circuit at 300 ng/mL of aTc was unimodal at two different times after induction. When the concentration of aTc was further increased (more than 1300 ng/mL), a bimodal population distribution was observed. An initial bimodal distribution of the gene expression for the MG::PR-8T circuit was observed at 1300 ng/mL of aTc and at 210 minutes after the induction (Additional File 1: Figure S4A). The expression distribution for MG::PR-8T remained bimodal over a period of induction times. This expression distribution began to show significant bimodality at two concentrations (1400, and 1500 ng/mL) of aTc at 210 and 330 minutes, with the relative fractions of bimodal fluorescence distributions dependent on aTc concentration. There are more transitions from a weak expression peak to a strong expression peak along the same point in time with increasing concentration of aTc. As a control, we also give the typical expression distributions of MG::PR-WT and MG::PR-1G at the concentration of 1500 ng/mL at 270 minutes (Additional File 1: Figure S3).

**Expression Distributions of the Non-Regulatory Gene Circuit in Microscopy**

The microscopy experiments of non-self-repressing fluorescence expression after aTc induction is shown in Additional File 1: Figure S7A. The expression distribution is unimodal. Unlike the MG::PR-8T circuit, the fluorescence intensities of the MG::PR-8T-P39K circuit without the inducer were strong. The MG::PR-8T-P39K circuit exhibits uniform Venus expression under all induction conditions (Additional File 1: Figure S7B).

**Single-Cell Fluorescence Trajectories for the MG::PR-8T Self-Repressor Circuit at Higher Inducer Concentration**

Many trajectories were taken from a time-lapse microscopy run at 1500 ng/mL of aTc. Each trajectory is formed from a single cell undergoing division and oscillation. The fluorescent images were periodically captured and recorded every 5 minutes. Additional file 1: Figure S8A shows a representative trajectory that exhibits significant low-high-low fluctuations: The tracked cell changed from a dark state to a brightened state, and then to a darkened state again as shown in Additional File 1: Figure S8B. Additional File 1: Figure S8C shows the other two representative trajectories that exhibit significant fluctuations. Moreover, as documented in Additional File 3: Movie S2, the cells exhibit the coexistence of the two cellular populations along with the cell divisions under prolonged induction times.

In some experiments, with an increase in the number of intracellular proteins, large numbers of proteins can produce metabolic burdens on the cells. These metabolic burdens will decrease the cell growth and division rate, while with little metabolic burden, the cells will grow quickly. This slower division is likely to result in protein accumulation in cells. Rapid cellular growth and division will dilute the proteins continuously, resulting in protein bimodal caused by growth rate (62). In order to explore the effects of cell growth rate, we conducted comparative experiments. The trajectories in Figure 4A and Additional File 1: Fig. S8 showed comparable growth rates in high expression state and in low expression state. It is possible that high expression cells in our study have not reached the threshold for significant metabolic burden to slow down the growth. From Fig. 4 and Additional File 1: Fig. S8, we noticed that the trajectories in our experiments exhibited significant fluctuations. This behavior is mostly likely from the change of the effective binding rate, rather than from the difference in growth rate since the growth rates are similar.

**Single-cell Fluorescence Trajectories for the MG::PR-8T Self-Repressor Circuit at a Lower Inducer Concentration**

Long-term single-cell fluorescence tracking was completed in constant cultivation environments containing 50 ng/mL of aTc with a constant temperature of 37 °C. Single-cell mean fluorescence intensities were captured every 8 minutes. The representative trajectory exhibits slight fluctuation as shown in Additional File 1: Figure S10A. As seen in Additional File 1: Figure S10B and Additional File 4: Movies S3, in the microscopy experiments at lower aTc concentration, the self-repressor circuit and associated function/dynamics was not significantly perturbed. Therefore, the change in cell population in regards to brightness is not as significant as that of the change in cell population at high aTc concentration.

**Data Analysis of the Time-Lapse Experiments**

**HMM analysis**

From the experimental real time trace of the gene expressions, one can infer the cell states as well as the rates of interconversion between them for statistics. We use HMM (Hidden Markov model) analysis of the data (63-65). HMM analysis of the data assumed a model of Markovian dynamics involving N discrete states. A HMM can be described by the probability transition between states at each step. There are many possible state-conditioned observation distributions, and we take the distribution to be the log-normal. We used the Baum-Welch algorithm, to obtain a maximum likelihood estimate of the HMM parameters. The analysis was performed on all the trajectories separately. For this analysis, we modified a freely-distributed MATLAB toolkit (<http://www.cs.ubc.ca/~murphyk/Software/HMM/hmm.html>) (63-65). Multiple random initial conditions were used to start the iterative HMM analysis to ensure convergence to the global minimum. The Baum-Welch algorithm was used to re-estimate the parameters at the end of each interaction.

Through the HMM analysis of the experimental real time trajectories, we identified two states and also the transition probabilities among those two states. The state probability evolution follows a master equation. Now we give a more detailed description for how switching or escape rates were obtained by HMM.

The master equation (with 2 states identified from the experimental trajectories from HMM model) can be written as

$$\frac{d}{dt}\left(\begin{matrix} P_{1} \\ P_{2} \end{matrix}\right)=\left(\begin{matrix} k_{11} & k_{12} \\ k_{21} & k_{22} \end{matrix}\right)\left(\begin{matrix} P_{1} \\ P_{2} \end{matrix}\right)=\left(\begin{matrix} -a & b \\ a & -b \end{matrix}\right)\left(\begin{matrix} P_{1} \\ P_{2} \end{matrix}\right)$$

Where P_1_ and P_2_ are the probabilities of the low expression state and the high expression state, respectively, k_ij_ (i,j=1,2) is the transition rate from state j to state i. We notice that for two states, the residence or waiting time of state i is the same as the transition time for state i to switch to state j. Therefore |k_11_| = |k_21_ |= a; |k_22_| =|k_12_| = b. Therefore, once a and b are known, we know the residence times of each state and the switching times from one state to another. In the following we give a detailed procedure how to obtain the switching times from the master equation based on the result of the HMM analysis.

We can write the solution of the master equation as follows with the initial condition P_1_(0)=1, P_2_(0)=0,

$$P_{1}\text{（t）=}\frac{b+ae^{-(a+b)t}}{a+b}$$

$$P_{2}\text{（t）=-}\frac{a(-1+e^{-(a+b)t})}{a+b}$$

Then the transition probability between the low expression state and the low expression state is

$$P_{11}=P_{1}\text{（}\delta\text{t）=}\frac{b+ae^{-(a+b)\delta t}}{a+b}$$

， where *δt* is the observation time for each time window, here *δt* = 5 min from our experiments.

With the initial condition P_1_(0)=0, P_2_(0)=1,

$$P_{1}\text{（t）=-}\frac{b(-1+e^{-(a+b)t})}{a+b}$$

$$P_{2}\text{（t）=}\frac{a+be^{-(a+b)t}}{a+b}$$

Then the transition probability between the high expression state and the high expression state is

$$P_{22}=P_{2}\text{（}\delta\text{t）=}\frac{a+be^{-(a+b)\delta t}}{a+b}$$

, where *δt* is the observation time for each time window, here *δt* = 5 min from our experiments.

According to the HMM analysis from the experimental real time trajectories of the expressions, the transition matrix is determined as

$$P=\left(\begin{matrix} P_{11} & P_{12} \\ P_{21} & P_{22} \end{matrix}\right)=\left(\begin{matrix} 0.977 & 0.023 \\ 0.037 & 0.963 \end{matrix}\right)$$

Therefore, we can obtain the transition rate:

a=0.004744 (1/min)

b=0.007631 (1/min)

| Switching  time  Next  state  Current state | High expression state | Low expression state |
| --- | --- | --- |
| High expression state | - | 92 min |
| Low expression state | 151 min | - |

The switching probability of the two expression states means that the transition probability of each observation in the experiment. In our experiment, we take photos every 5 minutes. So the time scale of the switching probability is 5 minutes, and we have given the time scale in the manuscript.

We use the same rate constants in the Gillespie simulations given in the section << Simple Mathematical Model Explaining the Self-Repressor Bimodal Distribution >>.

**Residence time and switching rate estimations**

Due to limitations in the measurement times, each state trajectory may or may not be measured for a sufficient amount of time. The residence times of the high expression state and the low expression state might be cut off by this measurement window.

To estimate the upper limit of the average residence time, we calculated the expected length of n consecutive states and the probability of residual time. The expected residual time for n consecutive states is calculated by the sum of the product of the probability and the remaining time of *N* consecutive states (*N*>n). The trajectories can be regarded as independent Bernoulli trials, and the probability can be calculated by

$$P_{N}=\prod_{i=1}^{N} p_{i}$$

where *P_N_* is the probability of *N* consecutive states, and *p_i_* is the transition probability of remaining in its own state.

The residual time can be given by

$$T=\sum_{N=n+1}^{\infty} P_{N}T_{N}$$

where *T_N_*  is the remaining time of *N* consecutive states.

The range of the average residence times of the high state is from 92 to 103 minutes, and the range of the average residence times of the low state is from 151 to 182 minutes.

**A Simple Mathematical Model Explaining the Self-Repressor Bimodal Distribution**

We simulated the stochastic dynamical process of the self-repression gene circuit under intrinsic fluctuations from the finite number of molecules in the cell. The dimer consists of two regulator proteins *TetR* where each can be bound on to the DNA promoter with rate ½ h n (n-1). Meanwhile, the dimer can be dissociated from the promoter with rates f *n, and the rate is the unbinding rate which is proportional to the number (n) of free *TetR*. (50)

where A^ij^ denotes the unbound (bound) state of the gene which will synthesize the *TetR* protein (i represents one regulator protein of the dimer binding and j represents another regulator protein of the dimer binding, 1 and 0 represent on and off of the genes respectively.). For the self-repression gene circuit, A^11^ denotes that the gene is completely switched “on” when the operator of the gene is active. A^00^ denotes that the gene is completely switched “off” when the operator of the gene is repressed. For the binding reaction, *TetR* dimer binds to DNA inhibition site to repress the gene expression, two *TetR* monomers combine to form a *TetR* dimer, and then the binding reaction can be simplified into a reaction between the gene and two *TetR* monomers. This leads to the quadratic dependence of the binding rate on the *TetR* concentrations. For the unbinding reaction, according to the rebinding model, the gene will react with the competitor. Due to the presence of the inducer, the conformation change of *TetR* will lead the *TetR* dimer convert into monomers (53), so the competitor can be chosen as *TetR* monomer. This leads to the linear dependence of the unbinding rate with respect to the free *TetR* concentrations.

We combined the transcription and translation steps for simplicity. Then, the transcription-translation step is described as follows:

where Ø is used to represent a protein sink or source. The source comes from gene activations while the sink comes from the protein degradations. g_ij_ is the protein synthesis rate when promoters are active or inactive for gene state A^ij^ respectively. When we added the inducers into the cells, the inducers aTc were found to bind with the regulator protein *TetR*. The resulting binding complex cannot bind effectively to the promoter, and therefore cannot have a significant influence on regulating gene transcription for synthesizing the protein. The binding complex of aTc and *TetR* may also degrade. This process can be expressed as follows:

where Ta denotes the binding complex of *TetR* and aTc. These binding complexes of aTc and *TetR* cannot occupy the binding site of the promoter. I_aTc_ and E_aTc_ denote the internal and the external inducer (in and out of the cells). The internal and external inducers can mutually diffuse with diffusion coefficient c. When the inducers are added into the solution, the inducers are gradually entered into the cell. In the experiment, we have maintained the steady flow supply of the aTc in the real time trace measurements. In other words, we tracked cells during their growth and division on a microscope with a FCS2 (Focht Chamber System 2, Bioptechs) system which provides aTc continuously to guarantee the cells growing in the right environments (continuous flow of adequate nutrients from fresh medium (M9) through the cells on agarose pad) and avoids potential issue of heterogeneity of the environments. Therefore the concentration of aTc outside the cell is kept constant. However, the aTc molecules inside the cell not only contribute to the free diffusion from in to the out of the cell but also to the binding to the *TetR*. Therefore the concentrations of the free aTc inside the cell and aTc outside of the cell are not equal. The steady state setup and the partition of aTc inside cell not only to the free but also to the binding to *TetR* will lead to the difference between the free inducer concentration inside the cell and inducer concentration outside the cell.

We define ω = f/k, which quantifies the ratio between the unbinding rate of *TetR* to the promoter and the speed of the *TetR* protein degradation. Likewise, the equilibrium constant Xeq = f/h measures the relative balance between dissociation and binding of *TetR* to the promoter. The binding is proportional to the concentration square of free TetR due to the dimer binding and unbinding is proportional to the concentration of free *TetR* (50). The free *TetR* can be an indicator of binding or unbinding. The binding of aTc and *TetR* will lead to a decrease in the number of free *TetR*. when the inducer is low, the binding and unbinding is relatively fast (adiabaticity), which leads to only one low expression distribution peak(32, 33). When the inducer concentration increases to sufficiently high values, the free *TetR* molecules will be fewer, the binding and unbinding rate will also be lower. This will increase the residence time of genes being at “on state” and “off state”. At the “on state”, more *TetR* will be generated, and then bind with the inducer, corresponding to the high expression peak. However, when the free *TetR* concentration is very low, the effect of unbinding (linear dependence on the free *TetR* concentration) is more significant compared to the effect of binding (quadratic dependence on free *TetR* concentration). As the aTc increases further, free *TetR* decreases. This reduces the unbinding rate more significantly than the binding rate. The less effective unbinding is expected to lead to more chances of genes being at off state. As a result, the lower expressions emerge (non-adiabaticity). According to the above mentioned setups and approach, we simulated the processes and reactions of the self-repressor gene regulation with inducers through the Gillespie algorithm. We obtained the probability distribution of the *TetR* protein concentration in different inducer concentrations. The simulated distribution of the proteins can be used for comparing the results to the experiments, measured by the fluorescence intensities of the *TetR* protein. As shown in Figure 3C, the simulated distribution of *TetR* protein is in reasonable agreements with the results from the experiments. We give all of rate constants as follows:

**Rate constants:**

Rate constants: g_11_=1000; g_00_=180; g_01_=g_10_=200; a=0.1; b=1; c=0.19; k_1_=3.5; k_2_=1.2; f=0.5; h=0.0083. (the unit of concentration is in number of molecules per cell and cell volume is equal to 1)

Or

Rate constants: g_11_=1510 nmol*L^-1^*h^-1^; g_00_=272 nmol*L^-1^*h^-1^; g_01_=g_10_=302 nmol*L^-1^*h^-1^; a=0.1 h^-1^; b=0.66 L*nmol^-1^*h^-1^; c=0.19h^-1^; k_1_=3.5 h^-1^; k_2_=1.2 h^-1^; f=0.75 nmol*L^-1^*h^-1^; h= 0.0055 L*nmol^-1^*h^-1^. (the unit of concentration is in nmol/L) (66, 67).

We assume cell volume is 1.1um^3^. For aTc, the molecular weight is 462.89.  A molecule of aTc per cell is about 0.7ng/mL or 1.51nmol/L.

We represent the chemical reactions with Master equation

$\begin{matrix} \frac{dP_{11}(n,m,s)}{dt}=-hn(n-1)P_{11}(n,m,s)+fnP_{10}(n-2,m,s)+fnP_{01}(n-2,m,s)+k_{1}[(n+1)P_{11}(n+1,m,s)-nP_{11}(n,m,s)] \\ +g_{11}[P_{11}(n-1,m,s)-P_{11}(n,m,s)]+b[(n+1)(s+1)P_{11}(n+1,m-1,s+1)-nsP_{11}(n,m,s)]+k_{2}[(m+1)P_{11}(n,m+1,s)-mP_{11}(n,m,s)] \\ +a[(m+1)P_{11}(n-1,m+1,s-1)-mP_{11}(n,m,s)]+cE_{aTc}(P_{11}(n,m,s-1)-P_{11}(n,m,s)]+c[(s+1)P_{11}(n,m,s+1)-sP_{11}(n,m,s)] \end{matrix}$ $\begin{matrix} \frac{dP_{01}(n,m,s)}{dt}=-\frac{1}{2}hn(n-1)P_{01}(n,m,s)+\frac{1}{2}h(n+1)(n+2)P_{11}(n+2,m,s)-fnP_{01}(n,m,s)+fnP_{00}(n-2,m,s)+k_{1}[(n+1)P_{01}(n+1,m,s) \\ -nP_{01}(n,m,s)]+g_{01}[P_{01}(n-1,m,s)-P_{01}(n,m,s)]+b[(n+1)(s+1)P_{01}(n+1,m-1,s+1)-nsP_{01}(n,m,s)]+k_{2}[(m+1)P_{01}(n,m+1,s)-mP_{01}(n,m,s)] \\ +a[(m+1)P_{01}(n-1,m+1,s-1)-mP_{01}(n,m,s)]+cE_{aTc}(P_{01}(n,m,s-1)-P_{01}(n,m,s)]+c[(s+1)P_{01}(n,m,s+1)-sP_{01}(n,m,s)] \end{matrix}$ $\begin{matrix} \frac{dP_{10}(n,m,s)}{dt}=-\frac{1}{2}hn(n-1)P_{10}(n,m,s)+\frac{1}{2}h(n+1)(n+2)P_{11}(n+2,m,s)-fnP_{10}(n,m,s)+fnP_{00}(n-2,m,s)+k_{1}[(n+1)P_{10}(n+1,m,s)-nP_{10}(n,m,s)] \\ +g_{10}[P_{10}(n-1,m,s)-P_{10}(n,m,s)]+b[(n+1)(s+1)P_{10}(n+1,m-1,s+1)-nsP_{10}(n,m,s)]+k_{2}[(m+1)P_{10}(n,m+1,s)-mP_{10}(n,m,s)] \\ +a[(m+1)P_{10}(n-1,m+1,s-1)-mP_{10}(n,m,s)]+cE_{aTc}(P_{10}(n,m,s-1)-P_{10}(n,m,s)]+c[(s+1)P_{10}(n,m,s+1)-sP_{10}(n,m,s)] \end{matrix}$ $\begin{matrix} \frac{dP_{00}(n,m,s)}{dt}=\frac{1}{2}h(n+1)(n+2)[P_{10}(n+2,m,s)+P_{01}(n+2,m,s)]-fnP_{00}(n,m,s)+k_{1}[(n+1)P_{00}(n+1,m,s)-nP_{00}(n,m,s)] \\ +g_{00}[P_{00}(n-1,m,s)-P_{00}(n,m,s)]+b[(n+1)(s+1)P_{00}(n+1,m-1,s+1)-nsP_{00}(n,m,s)]+k_{2}[(m+1)P_{00}(n,m+1,s)-mP_{00}(n,m,s)] \\ +a[(m+1)P_{00}(n-1,m+1,s-1)-mP_{00}(n,m,s)]+cE_{aTc}(P_{00}(n,m,s-1)-P_{00}(n,m,s)]+c[(s+1)P_{00}(n,m,s+1)-sP_{00}(n,m,s)] \end{matrix}$Where P_ij_(n,m,s) is the probability of gene state A^ij^, the variables *n*, *m* and *s* correspond to number of free *TetR*, Ta complex of *TetR* with aTc and inducer aTc. Because of the complexity of the master equation involving different gene states, we represent dynamics with moment equation.

The k^th^ moment is defined as: $\left\langle n_{ij}^{k}\right\rangle=\sum_{n,m,s} n^{k}P_{ij}(n,m,s)$

To get k^th^ order moment equation, multiply k^th^ and then sum over n,m,s on both side of master equation.

To obtain the deterministic equation for the average numbers of free *TetR*, inducer aTc and Ta complex of *TetR* with aTc molecules for stability and bifurcation analysis, we obtain the 0^th^ and 1^st^ order moment equation as following by applying self-consistent mean field approximation and Poisson ansatz.

$$\begin{matrix} \frac{dc_{11}}{dt}=-h(\frac{x_{1}^{2}}{c_{11}}-x_{1})+2fx_{2} \\ \frac{dc_{00}}{dt}=h(\frac{x_{2}^{2}}{c_{01}}-x_{2})-2fx_{3} \end{matrix}$$

$$\begin{matrix} \frac{dx_{1}}{dt}=-h(\frac{x_{1}^{3}}{c_{11}^{2}}-\frac{x_{1}^{2}}{c_{11}})+\frac{2f(x_{2}+2c _{01})^{2}}{c_{01}}+g_{11}c_{11}+ay_{1}-\frac{bx_{1}z_{1}}{c_{11}}-k_{1}x_{1} \\ \frac{dx_{2}}{dt}=\frac{h}{2}(\frac{x_{1}^{3}}{c_{11}^{2}}-\frac{3x_{1}^{2}}{c_{11}}+2x_{1})-\frac{h}{2}(\frac{x_{2}^{3}}{c_{01}^{2}}-\frac{x_{2}^{2}}{c_{01}})-\frac{fx_{2}^{2}}{c_{01}}+\frac{f(x_{3}+2c _{00})^{2}}{c_{00}}+g_{01}c_{01}+ay_{2}-\frac{bx_{2}z_{2}}{c_{01}}-k_{1}x_{2} \\ \frac{dx_{3}}{dt}=-h(\frac{x_{2}^{3}}{c_{01}^{2}}-\frac{3x_{2}^{2}}{c_{01}}+2x_{2})-\frac{2fx_{3}^{2}}{c_{00}}+g_{00}c_{00}+ay_{3}-\frac{bx_{3}z_{3}}{c_{00}}-k_{1}x_{3} \end{matrix}$$

$$\begin{matrix} \frac{dy_{1}}{dt}=-h\frac{y_{1}}{c_{11}}(\frac{x_{1}^{2}}{c_{11}}-x_{1})+\frac{2fy_{2}(x_{2}+2c _{01})}{c_{01}}+\frac{bx_{1}z_{1}}{c_{11}}-ay_{1}-k_{2}y_{1} \\ \frac{dy_{2}}{dt}=\frac{h}{2}\frac{y_{1}}{c_{11}}(\frac{x_{1}^{2}}{c_{11}}-x_{1})-\frac{h}{2}\frac{y_{2}}{c_{01}}(\frac{x_{2}^{2}}{c_{01}}-x_{2})-\frac{fx_{2}y_{2}}{c_{01}}+\frac{fy_{3}(x_{3}+2c _{00})}{c_{00}}+\frac{bx_{2}z_{2}}{c_{01}}-ay_{2}-k_{2}y_{2} \\ \frac{dy_{3}}{dt}=h\frac{y_{2}}{c_{01}}(\frac{x_{2}^{2}}{c_{01}}-x_{2})-\frac{2fx_{3}y_{3}}{c_{00}}+\frac{bx_{3}z_{3}}{c_{00}}-ay_{3}-k_{2}y_{3} \end{matrix}$$

$$\begin{matrix} \frac{dz_{1}}{dt}=-h\frac{z_{1}}{c_{11}}(\frac{x_{1}^{2}}{c_{11}}-x_{1})+\frac{2fz_{2}(x_{2}+2c _{01})}{c_{01}}+c(c_{11}E_{aTc}-z_{1})+ay_{1}-\frac{bx_{1}z_{1}}{c_{11}} \\ \frac{dz_{2}}{dt}=\frac{h}{2}\frac{z_{1}}{c_{11}}(\frac{x_{1}^{2}}{c_{11}}-x_{1})-\frac{h}{2}\frac{z_{2}}{c_{01}}(\frac{x_{2}^{2}}{c_{01}}-x_{2})-\frac{fx_{2}z_{2}}{c_{01}}+\frac{fz_{3}(x_{3}+2c _{00})}{c_{00}}+c(c_{01}E_{aTc}-z_{2})+ay_{2}-\frac{bx_{2}z_{2}}{c_{01}} \\ \frac{dz_{3}}{dt}=h\frac{z_{2}}{c_{01}}(\frac{x_{2}^{2}}{c_{01}}-x_{2})-\frac{2fx_{3}z_{3}}{c_{00}}+c(c_{00}E_{aTc}-z_{3})+ay_{3}-\frac{bx_{3}z_{3}}{c_{00}} \end{matrix}$$

Where, x­_1_=c_11_<n_11_>, x_2_=c_01_<n_01_>, x_3_=c_00_<n_00_>, y­_1_=c_11_<m_11_>, y_2_=c_01_<m_01_>, y_3_=c_00_<m_00_>, z­_1_=c_11_<s_11_>, z_2_=c_01_<s_01_>, z_3_=c_00_<s_00_>. The zero moments, c_11_,c_00_ and c_01_=(1-c_00_-c_11_)/2 measure the probability that the gene is in state A^ij^ . <n_ij_>, <m_ij_> and <s_ij_> correspond to the average number of free *TetR*, Ta complex of *TetR* with aTc and inducer aTc in gene state A^ij^ , respectively. Because the experimentally observable is x+y, we use W=x+y to denote the total *TetR* proteins (free *TetR* and Ta). We solved the moment equation and got the fixed point of all variables and the bifurcation diagram was given in Additional File 1: Figure S12.

**References**

1. Elowitz MB & Leibler S. A synthetic oscillatory network of transcriptional regulators. Nature. 2000;403:335-338.
2. Kobayashi H, Kærn M, Araki M, Chung K, Gardner TS, Cantor CR, & Collins JJ. Programmable cells: Interfacing natural and engineered gene networks. P Natl Acad Sci USA. 2004;101:8414-8419.
3. So LH, Ghosh A, Zong C, Sepúlveda LA, Segev R & Golding I. General properties of transcriptional time series in Escherichia coli. Nat Genet. 2011.43:554-U584.
4. Sprinzak D & Elowitz MB. Reconstruction of genetic circuits. Nature 2005;438:443-448.
5. Gardner TS, Cantor CR & Collins JJ. Construction of a genetic toggle switch in Escherichia coli. Nature. 2000;403:339-342.
6. Ackers GK, Johnson AD & Shea MA. Quantitative Model for Gene-Regulation by Lambda-Phage Repressor. P Natl Acad Sci-Biol. 1982;79:1129-1133.
7. Junker JP & van Oudenaarden A. Every Cell Is Special: Genome-wide Studies Add a New Dimension to Single-Cell Biology. Cell. 2014;157:8-11.
8. Krueger M, Scholz O, Wisshak S & Hillen W. Engineered Tet repressors with recognition specificity for the tetO-4C5G operator variant. Gene. 2007;404:93-100.
9. Shearwin K. Slow growth leads to a switch. Nat Chem Biol. 2009;5:784-785.
10. Pirchi M, Ziv G, Riven I, Cohen SS, Zohar N, Barak Y, & Haran G. Single-molecule fluorescence spectroscopy maps the folding landscape of a large protein. Nat Commun. 2011;2:493.
11. Bai F, Xu YC, Chen J, Liu QF, Gu JF, Wang XC, Ma JP, Li HL, Onuchic JN & Jiang HL. Free energy landscape for the binding process of Huperzine A to acetylcholinesterase. P Natl Acad Sci USA. 2013;110:4273-4278.
12. Ushikubo T, Inoue W, Yoda M, & Sasai M. Testing the transition state theory in stochastic dynamics of a genetic switch. Chem Phys Lett. 2006;430:139-143.
13. Nevozhay D, Adams RM, Van Itallie E, Bennett MR, Balázsi G.. Mapping the environmental fitness landscape of a synthetic gene circuit. PLOS Comput Biol. 2012;8:e1002480.
14. Murphy KF, Adams RM, Wang X, Balazsi G. & Collins JJ. Tuning and controlling gene expression noise in synthetic gene networks. Nucleic Acids Res. 2010;38: 2712-2726.

| **Primer** | **Sequence and Restriction Enzymes** |  |
| --- | --- | --- |
| *TetR*-F: | 5’-CAGGGTACCATGTCCAGATTAGATAAAAGTAAAG-3’ | *Kpn*I |
| Venus-R: | 5’-CATTCTAGATTACTTGTACTG-3’ | *Xba*I |
| *TetR* -Linker-F | 5’-GTGAAAGTGGGTCTAAGGGTGGGCGCGC-3’ |  |
| *TetR* -Linker-R | 5’-CCCACCCTTAGACCCACTTTCACATTTAAG-3’ |  |
| Ptet-8T-F | 5’-CAGCTCGAGTCCCTATCAGTGATAGATATTGACATCCCTATCAGTGATAGATATACTG-3’ | *Xho*l |
| P39K-F | 5’-CAGAAGACATTGTATTGGCATG-3’ |  |
| P39K-R | 5’-CAATGTCTTCTGCTCTACACCTAG-3’ |  |
| Seq-pZE-F | 5’-CATGAGCGGATACATATTTG-3’ |  |
| Seq-pZE-R | 5’-TCAGGAGAGCGTTCACCGAC-3’ |  |
| Seq-pAH-F | 5’-ACGCCCGCCATAAACTG-3’ |  |
| Seq-pAH-R | 5’-GAAATACCCAGCCTCGC-3’ |  |
| P1 | 5’-GGCATCACGGCAATATAC-3’ |  |
| P4 | 5’-TCTGGTCTGGTAGCAATG-3’ |  |
| P2 | 5’-ACTTAACGGCTGACATGG-3’ |  |
| P3 | 5’-ACGAGTATCGAGATGGCA-3’ |  |

**Table S1: PCR primers used in this study.** All primer sequences are listed in 5' to 3' orientation. Recognition sites for restriction enzymes are underlined.

The *TetR* fragment was produced by PCR using primers *TetR*-F and *TetR*-Linker-F from the vector Plasmid pBR322 (Invitrogen™), while the linker-Venus fragment was produced by PCR using primers *TetR* -Linker-F and Venus-R from the chromosome of *E. coli* strain SX4. The *TetR* fragment and the linker-Venus fragment contain an overlap sequence at 3' and 5' ends, respectively, of which the two ends are fused by DNA polymerase, and have produced the *TetR*-Venus fused fragment by PCR using 'nested' primers *TetR*-F and Venus-R. The different forward primers with the *Xho*I site and the same reverse primer with the *Xba*I site were used in a series of promoter constructions (primers that constructed the MG::PR-8T circuit are shown in Table S1，others are not shown). Restriction sites within primers are indicated with underlining. We utilized P39K-F and P39K-R PCR primers for site-directed mutagenesis to construct pAH150-P*LtetO*-1-8T-*TetR*-P39K-Venus. Seq-pZE-F and Seq-pZE-R were used as the sequencing primers for constructing pZE11-P*LtetO-1-TetR*-Venus, while Seq-pAH-F and Seq-pAH-R were used for pAH150-P*LtetO-1-TetR*-Venus and a series of self-repressing plasmids. P1, P2, P3, and P4 primers were used in PCR tests and sequencing for single-copy chromosomal integration.

| Mass concentration | 300  ng/mL | 700  ng/mL | 1000  ng/mL | 1200  ng/mL | 1300  ng/mL | 1400  ng/mL | 1500  ng/mL |
| --- | --- | --- | --- | --- | --- | --- | --- |
| Molar concentration | 6.48*10^-10^  mol/mL | 1.51*10^-9^  mol/mL | 2.16*10^-9^  mol/mL | 2.59*10^-9^  mol/mL | 2.80*10^-9^  mol/mL | 3.02*10^-9^  mol/mL | 3.24*10^-9^  mol/mL |

**Table S2. The corresponding relationship between the mass concentration and the molar concentration**

| **Parameter** | **Value in model** | **Source** | **Description** |
| --- | --- | --- | --- |
| g_11_ | 1000 molecules *h^-1^ or 1510 nmol*L^-1^*h^-1^ | 978 molecules*h^-1^ for *TetR*(67) | Synthesis rate of *tetR* when the gene is completely switched "on" |
| g_00_ | 180 molecules *h^-1^ or 272 nmol*L^-1^*h^-1^ | 0 molecules*h^-1^ for *TetR* (67) | Synthesis rate of *tetR* when the gene is completely switched "off" |
| g_01_  g_10_ | 200 molecules *h^-1^ or 302 nmol*L^-1^*h^-1^ | 0 molecules*h^-1^ for *TetR* (67) | Synthesis rate of *tetR* when a gene is switched "off" and the other is "on" |
| a | 0.1 h^-1^ or 0.1 h^-1^ | 0 h^-1^ for *TetR* (67) | Decomposed rate of the binding complex of *TetR* and aTc |
| b | 1 molecules^-1^*h^-1^ or 0.66 L*nmol^-1^*h^-1^ | 1.5 molecules^-1^*h^-1^ for *TetR* (67) | Synthesis rate of the binding complex of *TetR* and aTc |
| c | 0.19 h^-1^ or 0.19 h^-1^ | 0.9242 h^-1^ for *TetR* (67) | The internal and external inducers aTc diffusion coefficient |
| k_1_ | 3.5 h^-1^ or 3.5 h^-1^ | 2.1 h^-1^ for *TetR* (66) | Degradation rate of the protein *TetR* |
| k_2_ | 1.2 h^-1^ or 1.2 h^-1^ | 1 h^-1^ for *TetR* (66) | Degradation rate of the binding complex of *TetR* and aTc |
| f | 0.5 molecules *h^-1^ or 0.75 nmol*L^-1^*h^-1^ | 3.46 molecules*h^-1^ for *TetR* (67) | Dissociated rate of the *tetR* dimer from the promoter |
| h | 0.0083 molecules^-1^*h^-1^ or 0.0055 L*nmol^-1^*h^-1^ | 0.06 molecules^-1^*h^-1^ for *TetR* (67) | Binding rate of the *tetR* dimmer |
| f/h in Figure 3C | 60 | - |  |
| f/h in Additional File 1: Fgure S16 | 120 | - |  |
| f/h in Additional File 1: Figure S17 | 1 | - |  |

**Table S3. Rate constants in mathematical model.** We have listed the parameters used in the mathematical model. The unit of concentration is in number of molecules per cell or in nmol/L. We assume cell volume is 1.1um^3^. For aTc, the molecular weight is 462.89.  A molecule of aTc per cell is about 0.7ng/mL or 1.51nmol/L.


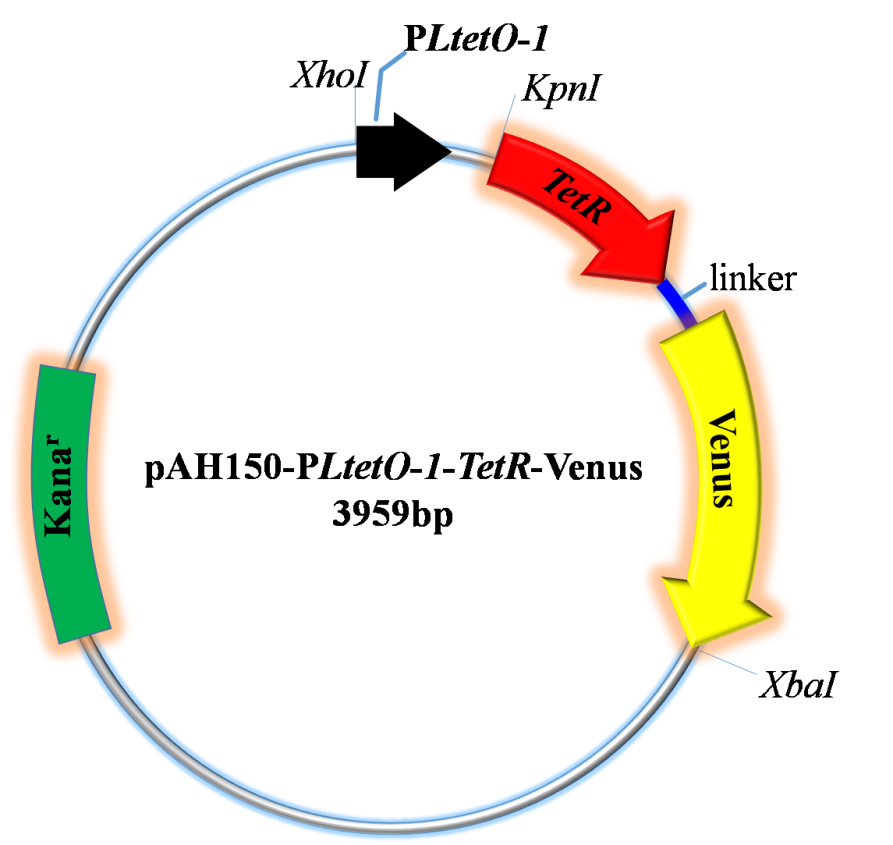


**Figure S1. The recombinant plasmid map containing the self-repressing circuit.** The self-repressing circuit is built on a low copy number CRIM plasmid with pAH150. It contains the *TetR*-Venus gene under the transcriptional control of the P*LtetO-1* promoter as a self-repressing circuit for integration in a MG1655 chromosome.


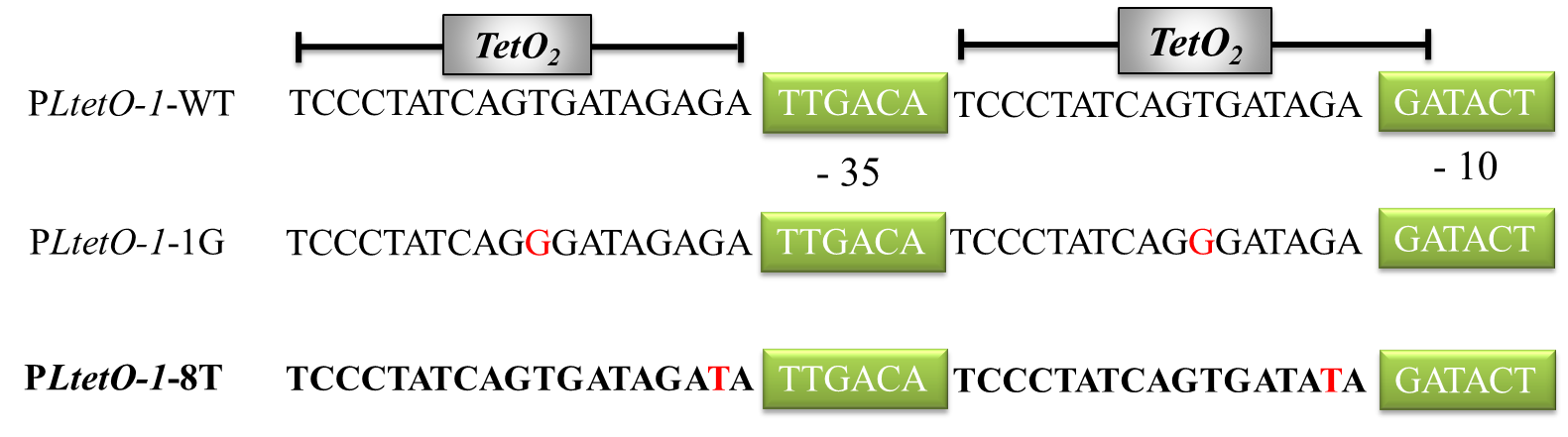


**Figure S2. Diagram of synthetic circuit constructs.** The architecture of each promoter is shown with two functional operators (*TetO_2_*) and -10 and -35 boxes (green). The name of each promoter is indicated to its left in the diagram. Nucleotide sequences of wild type and mutant Tn10-encoded *TetO_2_* are given on the left of the figure (red letters indicate mutated bases).


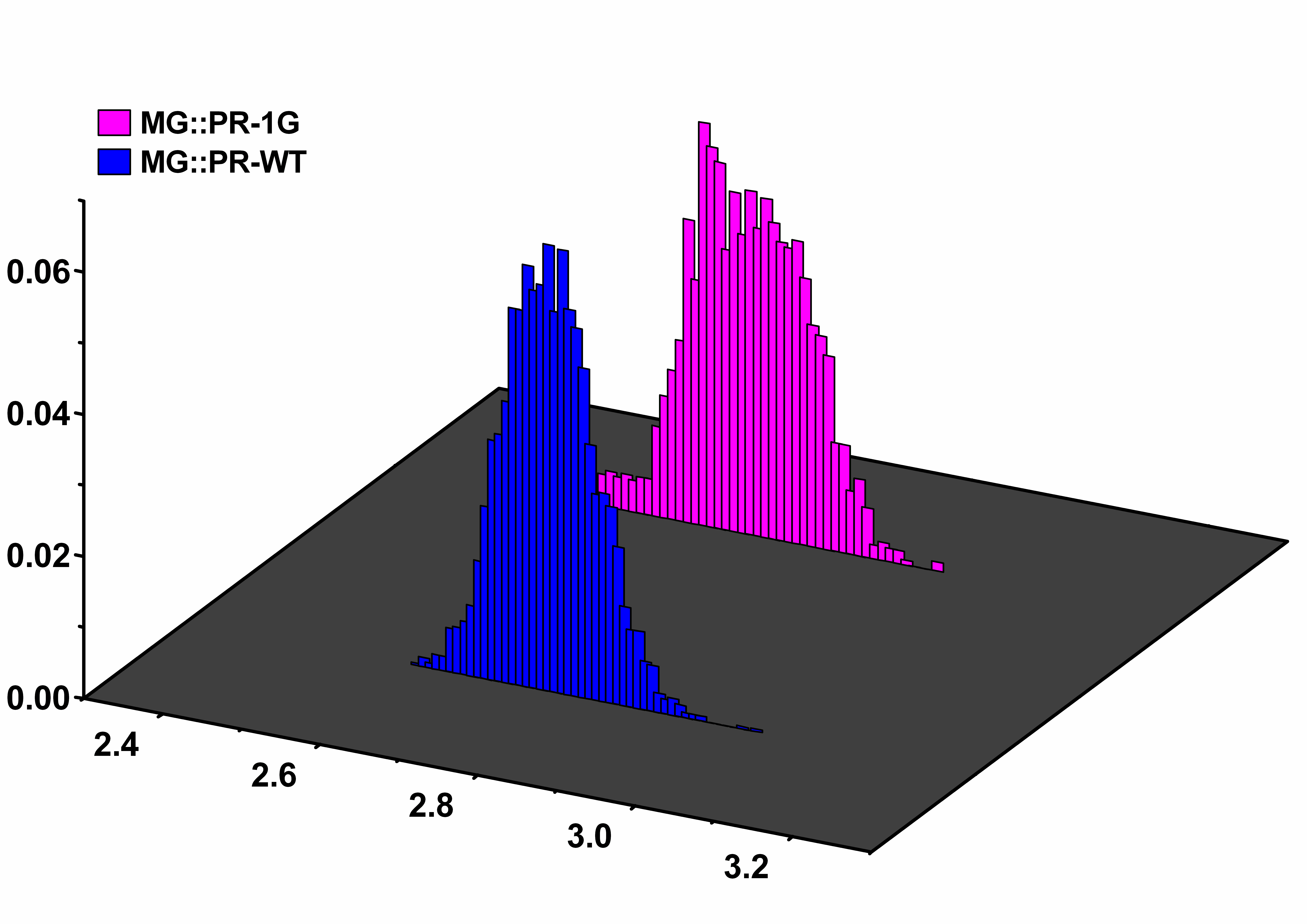


**270 min**

**1500 ng/ml**

**Figure S3. The distributions of mean fluorescence intensity of MG::PR-WT and MG::PR-1G induced by 1500 ng/mL aTc concentrations at 270 minutes.**

**210 min**


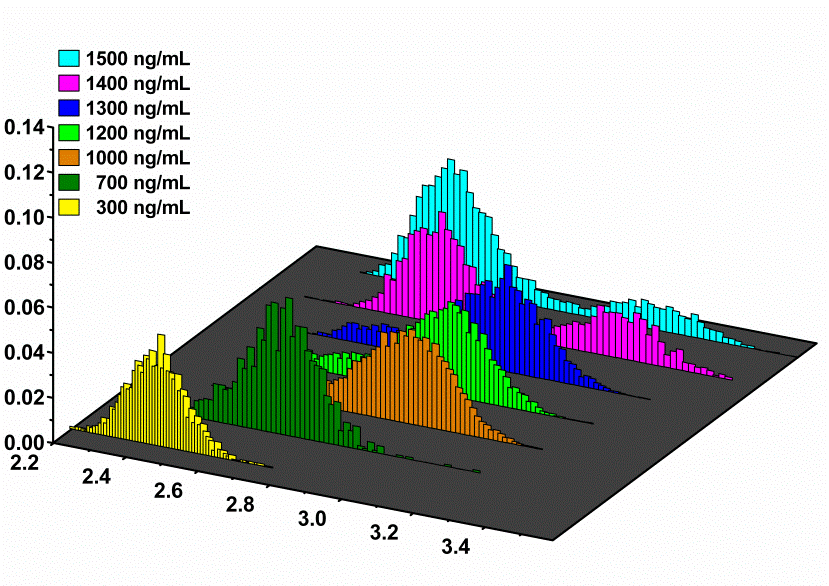


**330 min**


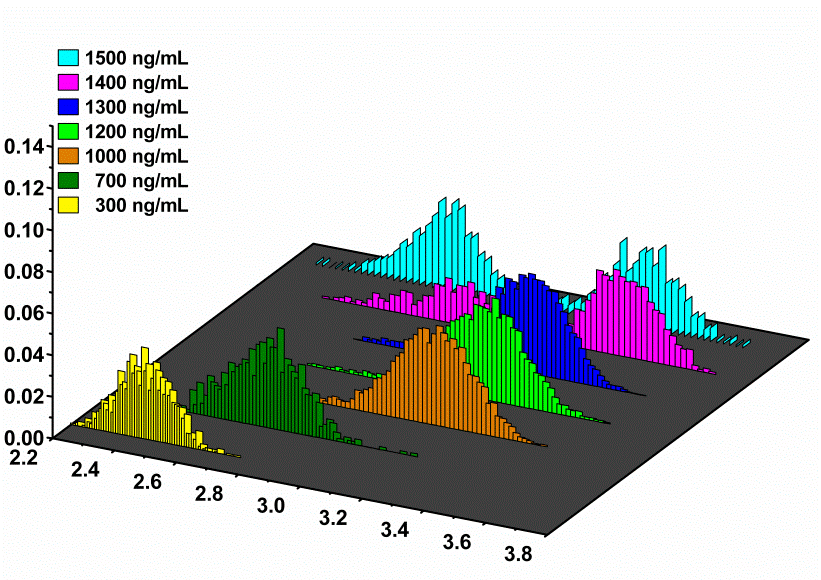


**Figure S4. The distributions of mean fluorescence intensity of MG::PR-8T** **induced by various aTc concentrations at different times.** (A) and (B) show the expression distribution of MG::PR-8T circuit was measured by single-cell fluorescence microscopy at 210 and 330 minutes after the induction at all inducer concentrations(300, 700, 1000, 1200, 1300, 1400, and 1500 ng/mL ). Seven color histograms represent different inducer concentrations. The x-axis represents the logarithm of mean fluorescence intensity, which is equivalent to the total fluorescence divided by cell area (for every cell). The data was analyzed using microscopy images of cells with MATLAB.

**B**

**A**


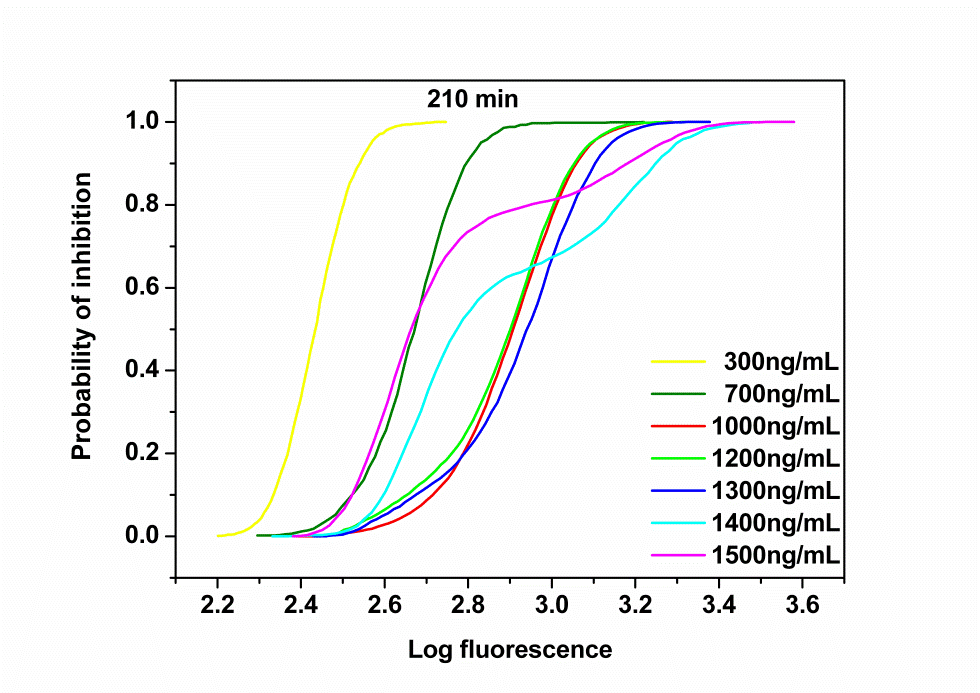

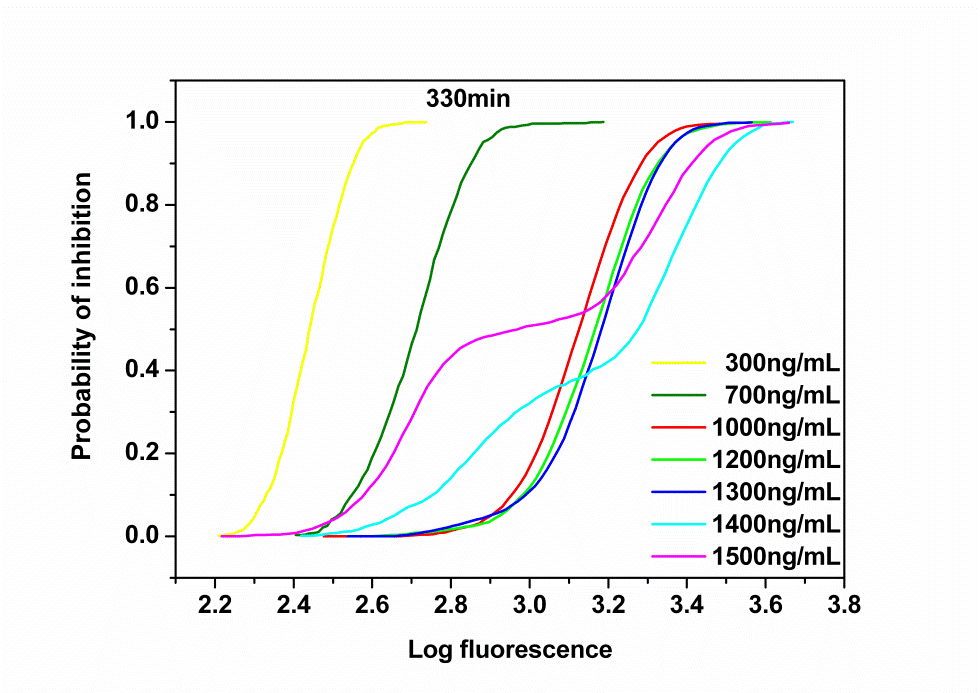


**Figure S5. Total probability of the DNA in the bound state as a function of the mean fluorescence. (**A) and (B) show the probability of inhibition curves of the MG::PR-8T circuit at 210 and 330 minutes after the induction at all inducer concentrations(300, 700, 1000, 1200, 1300, 1400, and 1500 ng/mL ). Seven color histograms represent different inducer concentrations. The inhibition curves were calculated by the ratio of the cells with a fluorescence intensity lower than a certain value to the number of total samples.

**A**


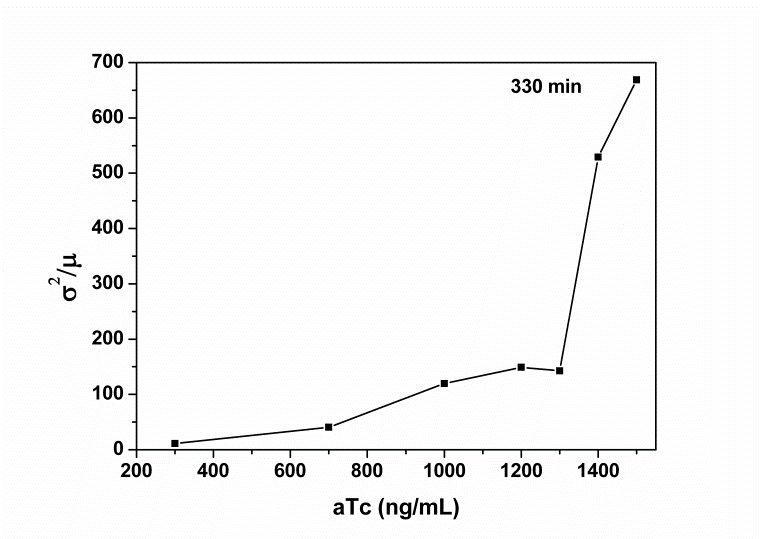


**B**


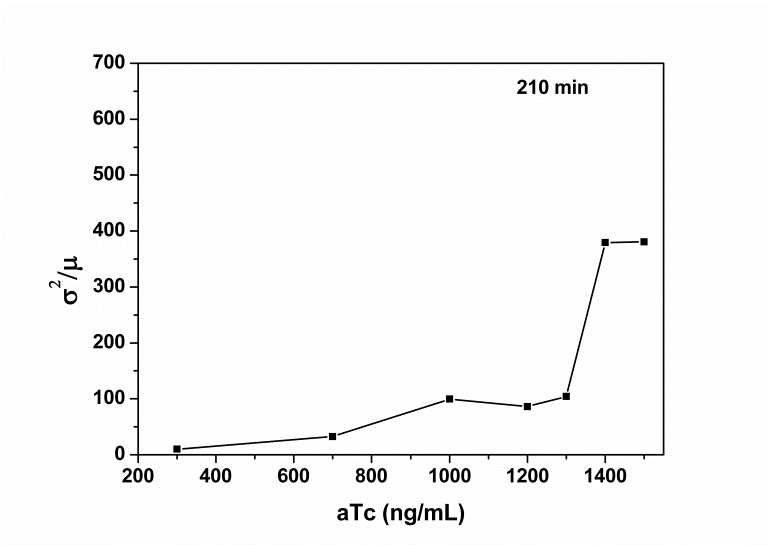

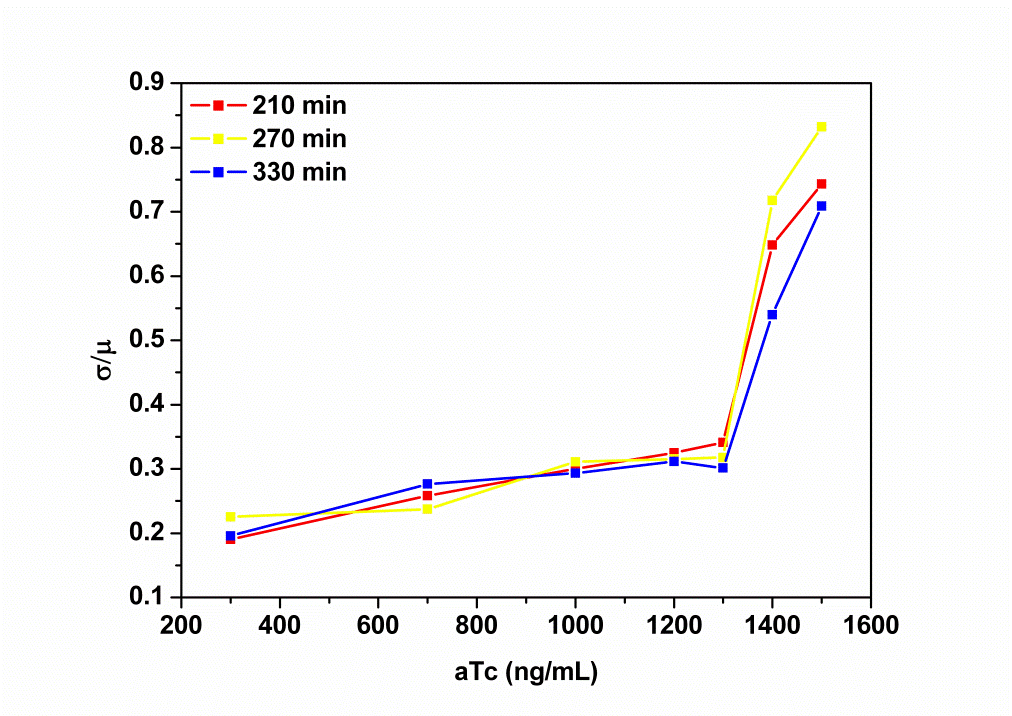


**Figure S6. Fano factor curves and the overall coefficient of variation (CV) curves of the self-repressing gene circuit.** (A) The curves respectively represent Fano factor curves of MG::PR-8T circuit at 210 and 330 minutes after the induction at different inducer concentrations. The Fano factor is defined as σ2/μ, where σ and μ are the standard deviation and the mean of the probability distribution. (B) Dose-response of the CV of *TetR*-Venus expression for the self-repressing gene circuit. Red, yellow, and blue curves respectively represent the CV of the MG::PR-8T circuit at 210, 270 and 330 minutes after induction at different inducer concentrations. CV is defined as σ/μ, where σ and μ are the standard deviations and the mean of the probability distribution.

**Probability**

**Log fluorescence**

**A**


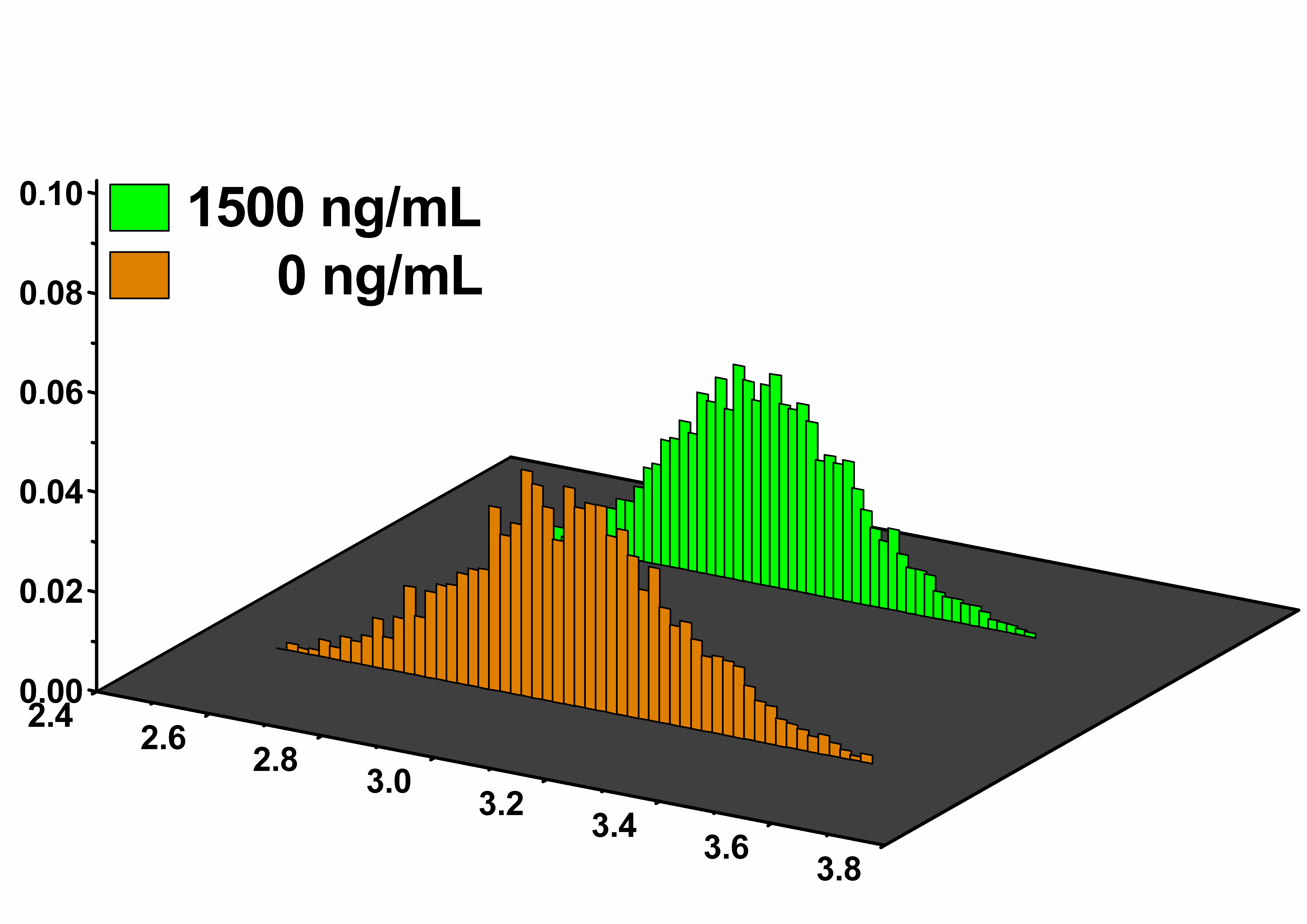


**B**

**aTc 1500 ng/mL**

**aTc 0 ng/mL**


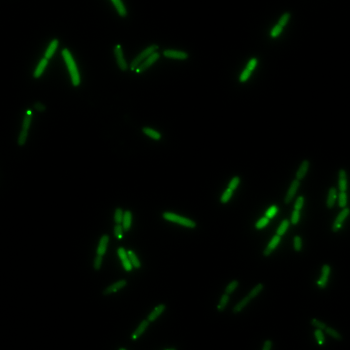

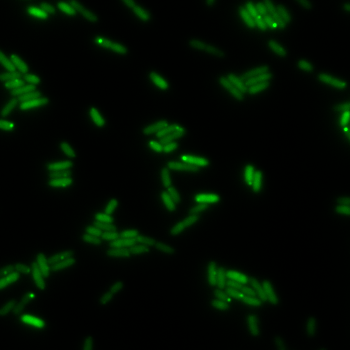


**Figure S7. Expression distributions of MG::PR-8T-P39K induced by various aTc concentrations at different times.** We selected 0 and 1500 ng/mL aTc inducer concentrations and collected the data. The x-axis represents the logarithm of mean fluorescence intensity, which is equivalent to total fluorescence divided by cell area (for every cell). (A) The expression distribution of the MG::PR-8T-P39K circuit is always unimodal. (B) The fluorescence images of the corresponding measurements (0 and 1500 ng/mL of aTc at 270 minutes after the induction).


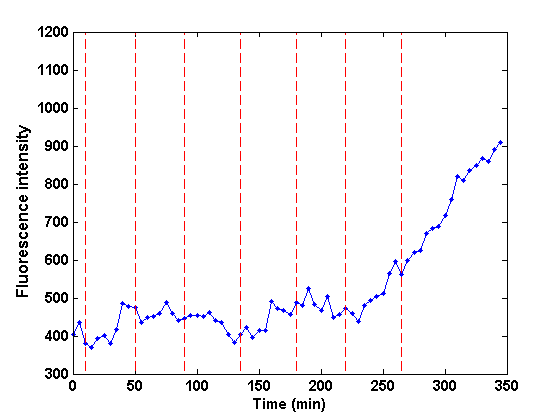

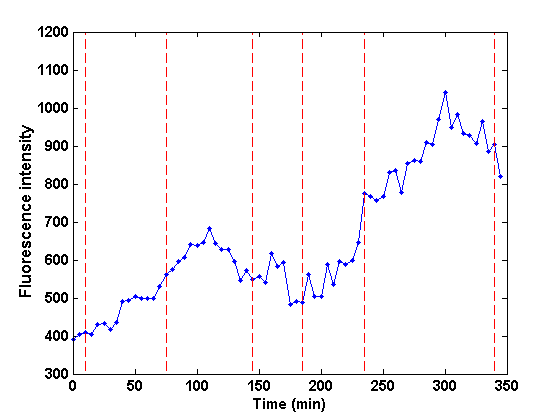


**A**

**B**

**C**


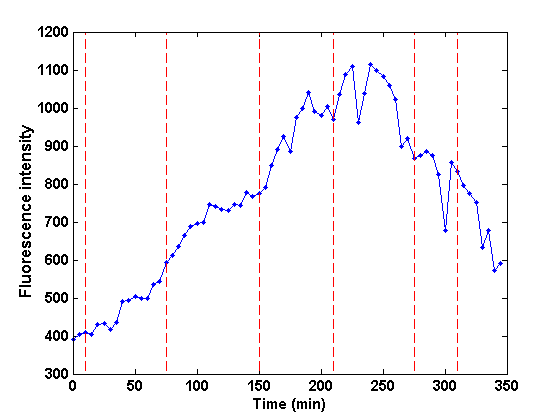

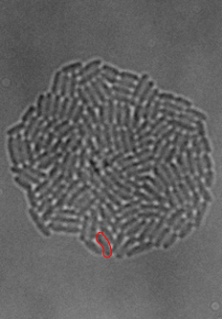

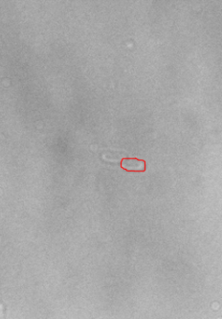

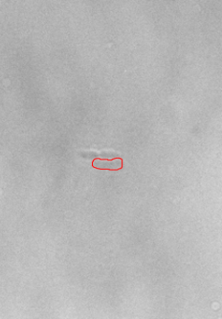

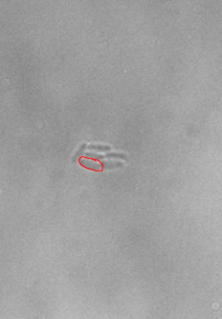

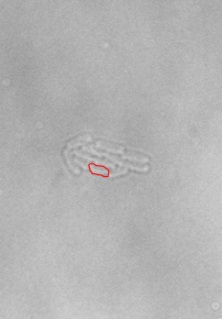

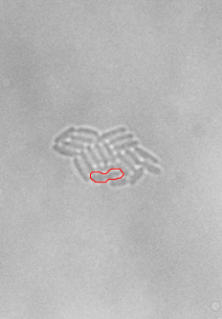

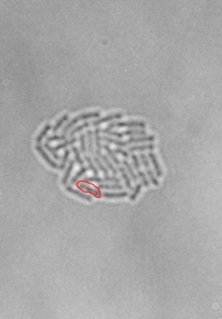

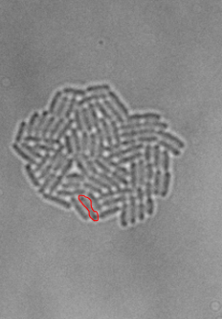

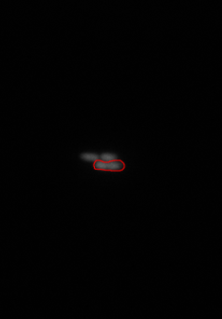

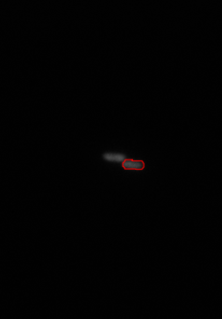

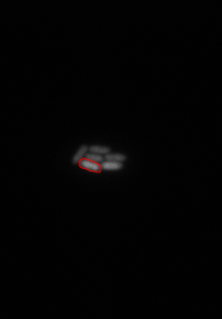

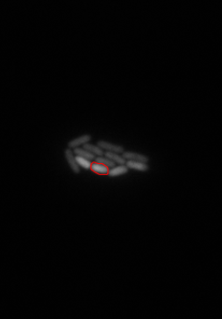

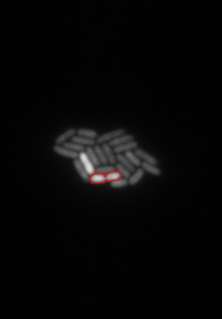

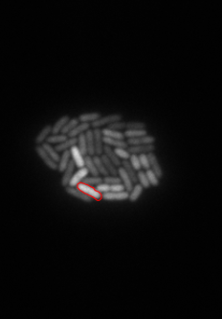

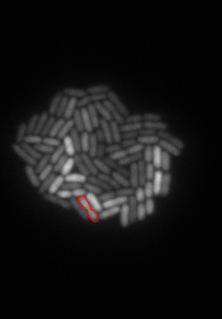

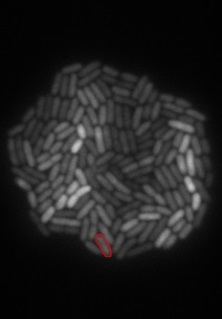


**0 min**

**50 min**

**100 min**

**150 min**

**200 min**

**250 min**

**300 min**

**350 min**

**Figure S8. The representative trajectories collected at 1500 ng/mL of aTc for the MG::PR-8T strain.** Long-term single-cell fluorescence tracking was completed at 1500 ng/mL of aTc. (A) A representative single-cell fluorescence trajectory induced with 1500 ng/mL of aTc. Points represent experimental fluorescence values. Red vertical dashed lines demarcate cell divisions. (B) The bright field and fluorescent field images in the time-lapse experiment corresponding to Fig. S8A. The cells corresponding to the fluorescence trajectory are marked with red circles. The average of bacteria mean fluorescence intensity is 591 and the average cell cycle time is 47 minutes in this micro-colony. (C) Two trajectories with different trends randomly selected from Fig. S8B.


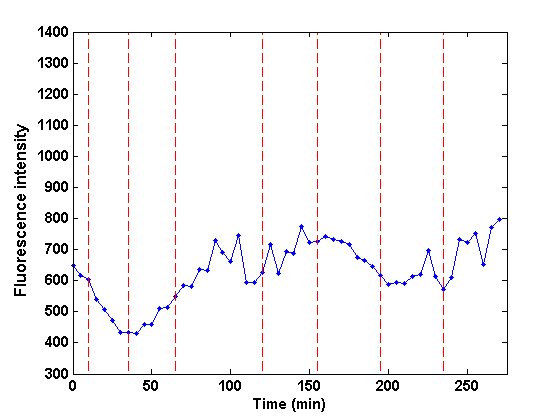


**A**

**B**


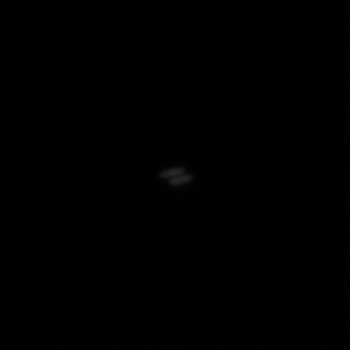

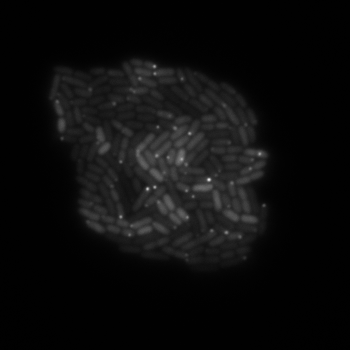

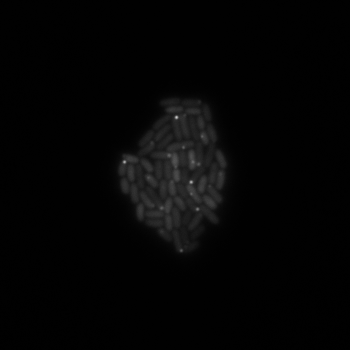

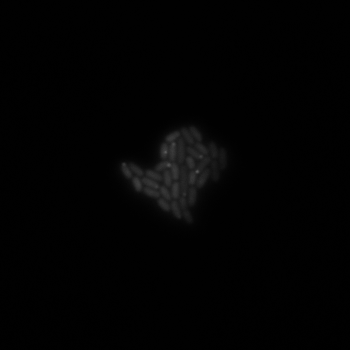

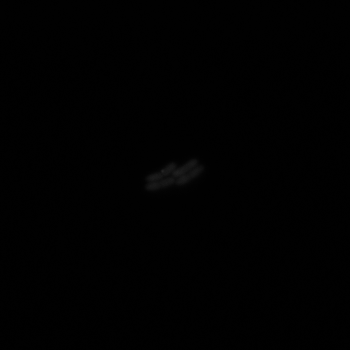

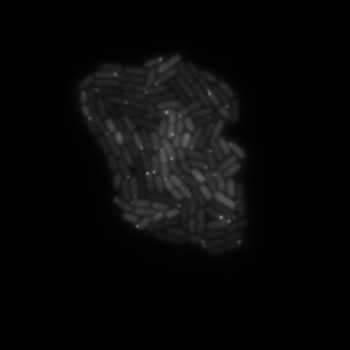

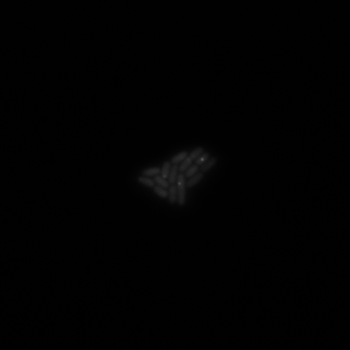

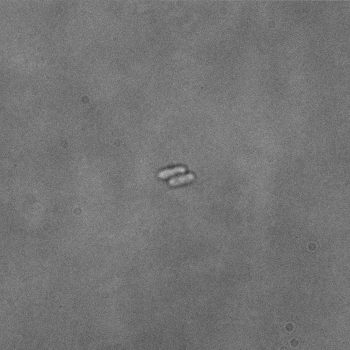

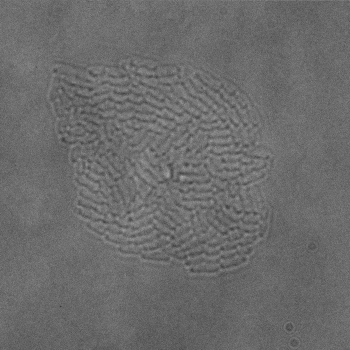

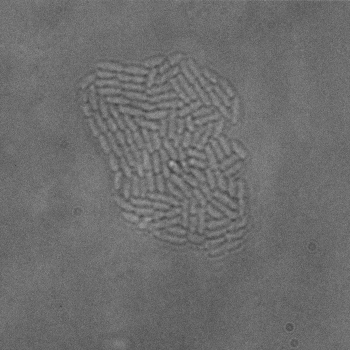

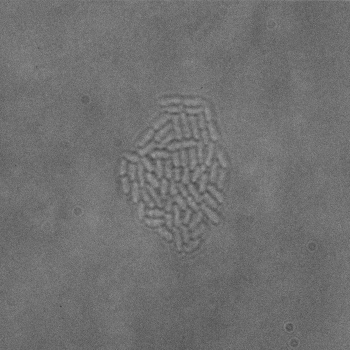

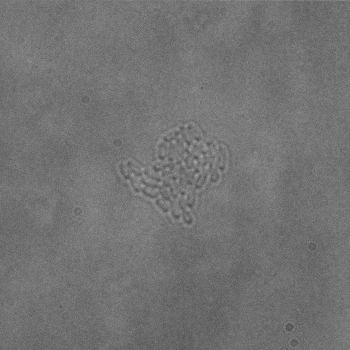

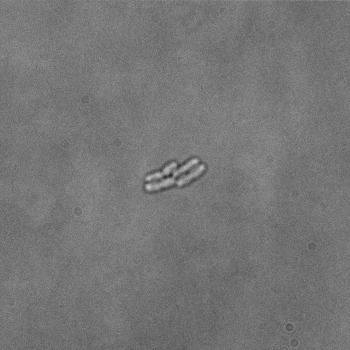

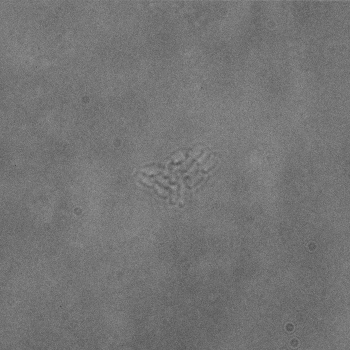


**0 min**

**50 min**

**100 min**

**150 min**

**200 min**

**250 min**

**275 min**

**275 min**

**0 min**

**Figure S9. The fluorescence images of MG::PR-8T-P39K in a time-lapse experiment with aTc of 1500 ng/mL.** (A) A representative single-cell fluorescence trajectory induced with 1500 ng/mL of aTc. Points represent experimental fluorescence values. Red vertical dashed lines demarcate cell divisions. (B) The bright field and fluorescent field images in the time-lapse experiment. The experiment was conducted at all test conditions as a control group to observe the growth state of bacteria. The average of bacteria mean fluorescence intensity is 910 and the average cell cycle time is 38 minutes in this micro-colony.

**B**


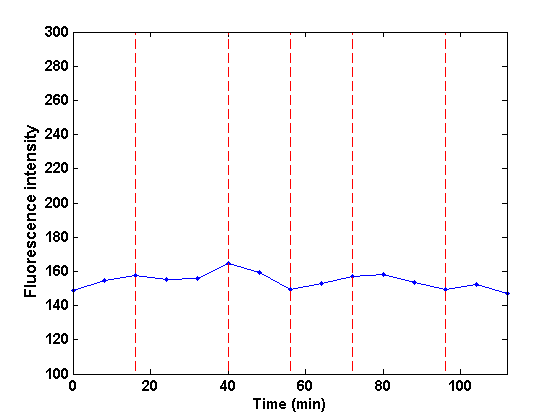


**A**

**(a)**

**(b)**


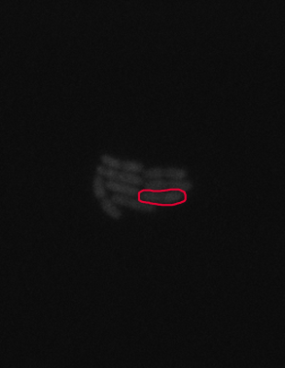

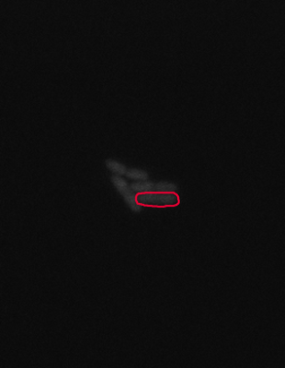

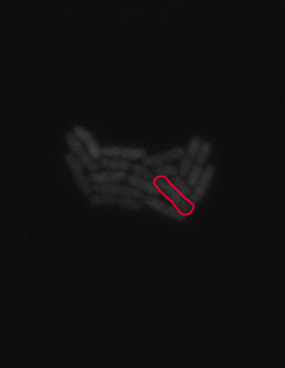

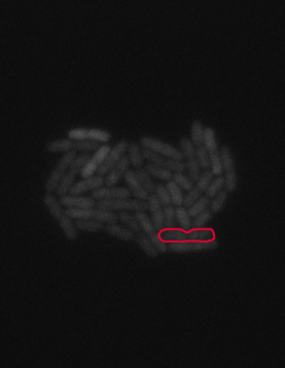

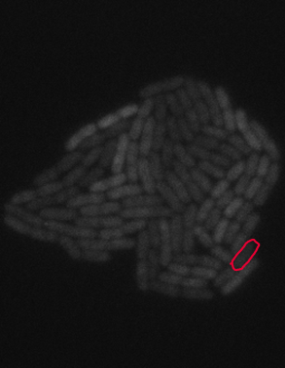

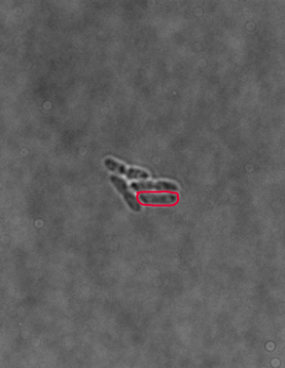

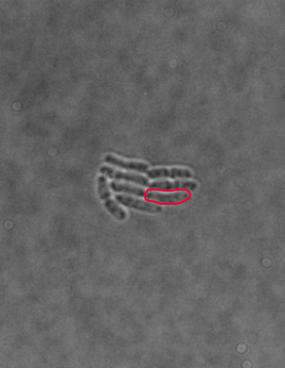

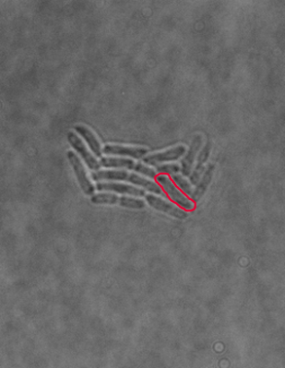

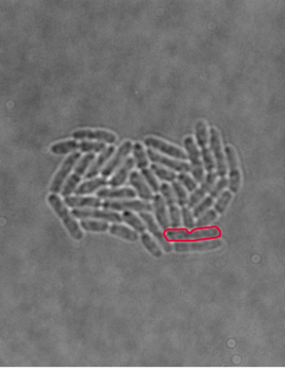

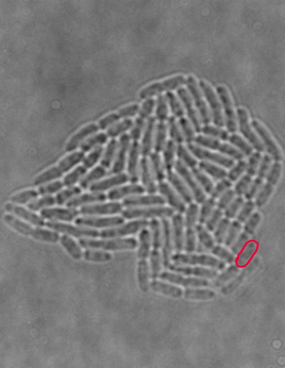


**0 min**

**24 min**

**48 min**

**72 min**

**96 min**

**Figure S10. A representative trajectory collected at 50 ng/mL of aTc for the MG::PR-8T strain.** Long-term single-cell fluorescence tracking was completed in M9 media containing 50 ng/mL of aTc. Media were infused to the micro-channel at 1-2 µL/s with the temperature maintained at 37°C. The cells were tracked by fluorescence microscopy and analyzed using MATLAB software. (A) A representative single-cell fluorescence trajectory induced with 50 ng/mL of aTc. Points represent experimental fluorescence values. Red vertical dashed lines demarcate cell divisions.

(B) The bright field and fluorescent field images in the time-lapse experiment corresponding to Figure S10A. The cells corresponding to the fluorescence trajectory are marked with red circles.

**B**

**A**

**c**


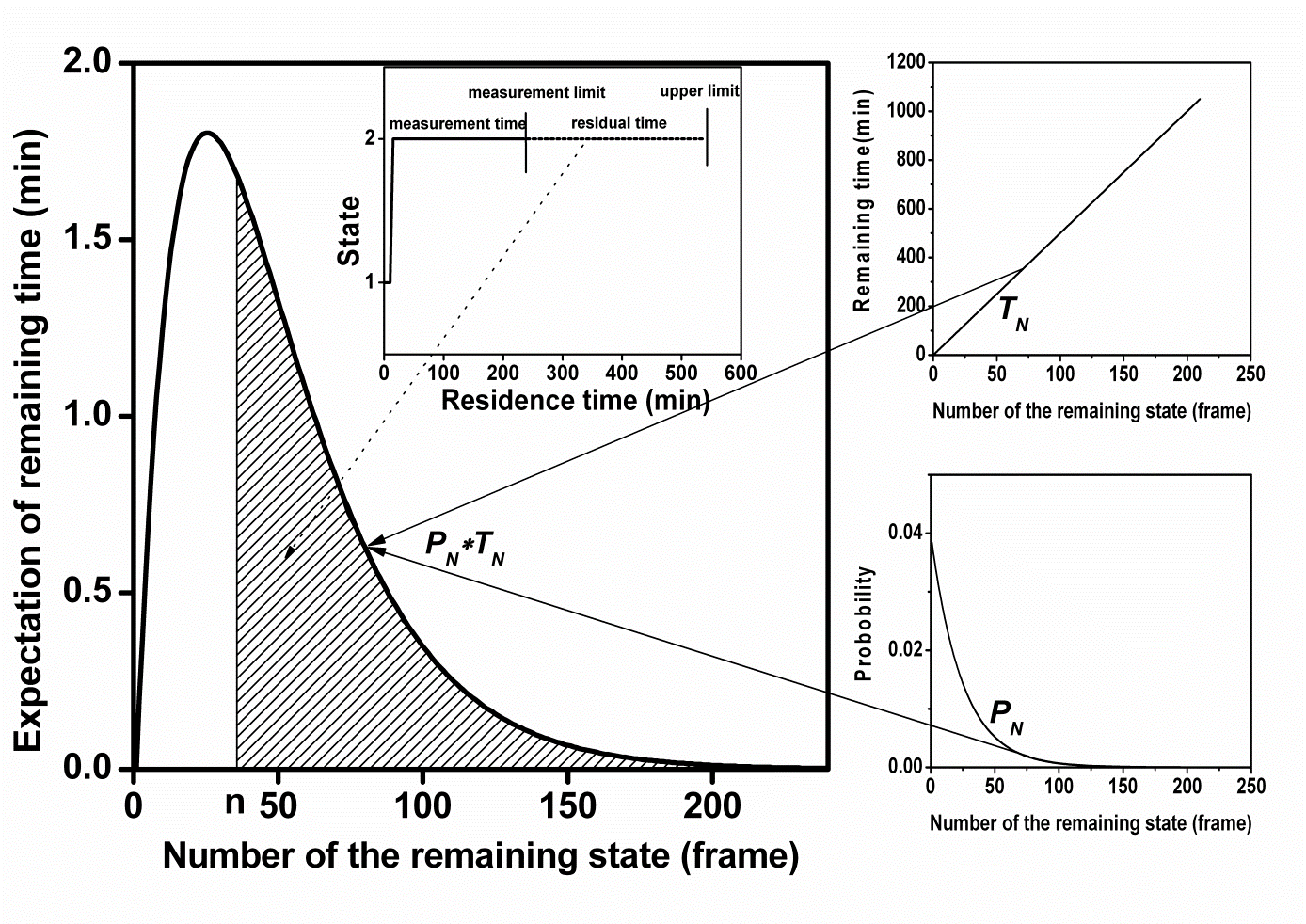


**Figure S11. The schematic diagram of calculating the average residence time (each frame lasts 5 minutes).** (A) The x-axis represents the number of the remaining states in frames and the y-axis represents the expectation of the remaining time. The inset image on the upper right shows the process of the state exchanges. The dash associated with the area of the shadow is the possible residual time, which may not be fully observed within the test time limit. To estimate the upper limit of the average residence time, we calculate the expected time of n consecutive states and the probability of residual time. The expected residual time for n consecutive stated is calculated by the sum of the product of the probability and all the remaining time of *N* consecutive states (*N*>n). The residual time can be given by:

$$T=\sum_{N=n+1}^{\infty} P_{N}T_{N}$$

where *T_N_* is the remaining time of *N* consecutive states. The average residence time is calculated by the sum of the measurement time and the residual time.

(B) The x-axis represents the number of remaining states and the y-axis represents the remaining time.

(C) The x-axis also represents the number of remaining states, and the y-axis represents the probability of the remaining time in the survival state. The trajectories can be regarded as independent Bernoulli trials, and the probability can be calculated by

$$P_{N}=\prod_{i=1}^{N} p_{i}$$

where *P_N_* is the probability of *N* consecutive states, and *p_i_* is the transition probability of the cell remaining in its own state.


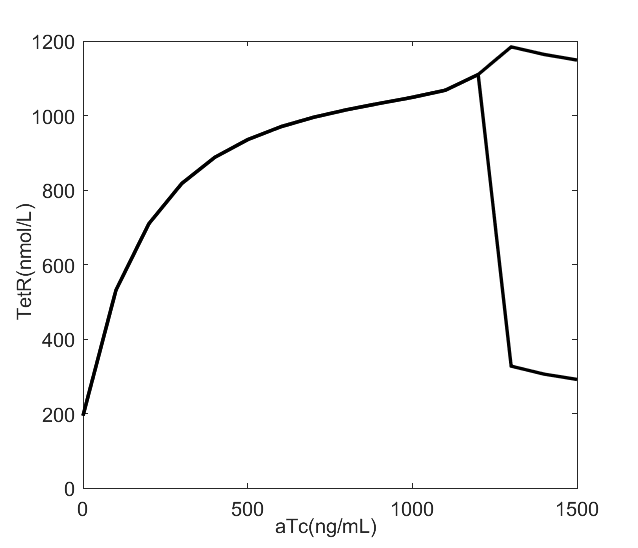


Figure S12. The bifurcation diagram in total number of *TetR* (in nmol/L) molecules and aTc (in ng/mL) molecules. When the concentration of the inducer aTc molecules is less than 1200 ng/mL, it corresponds to that the number of *TetR* molecules is almost equally populated in gene on and off states A^11^ and A^01^. The peaks of the on and off gene states are merged to one. When the number of aTc molecules is more than 1200 ng/mL, it corresponds to that the number of *TetR* molecules is significantly differently populated in gene on and off states A^11^ and A^01^. The on and off gene states are separated and the associated distribution has two clear peaks.


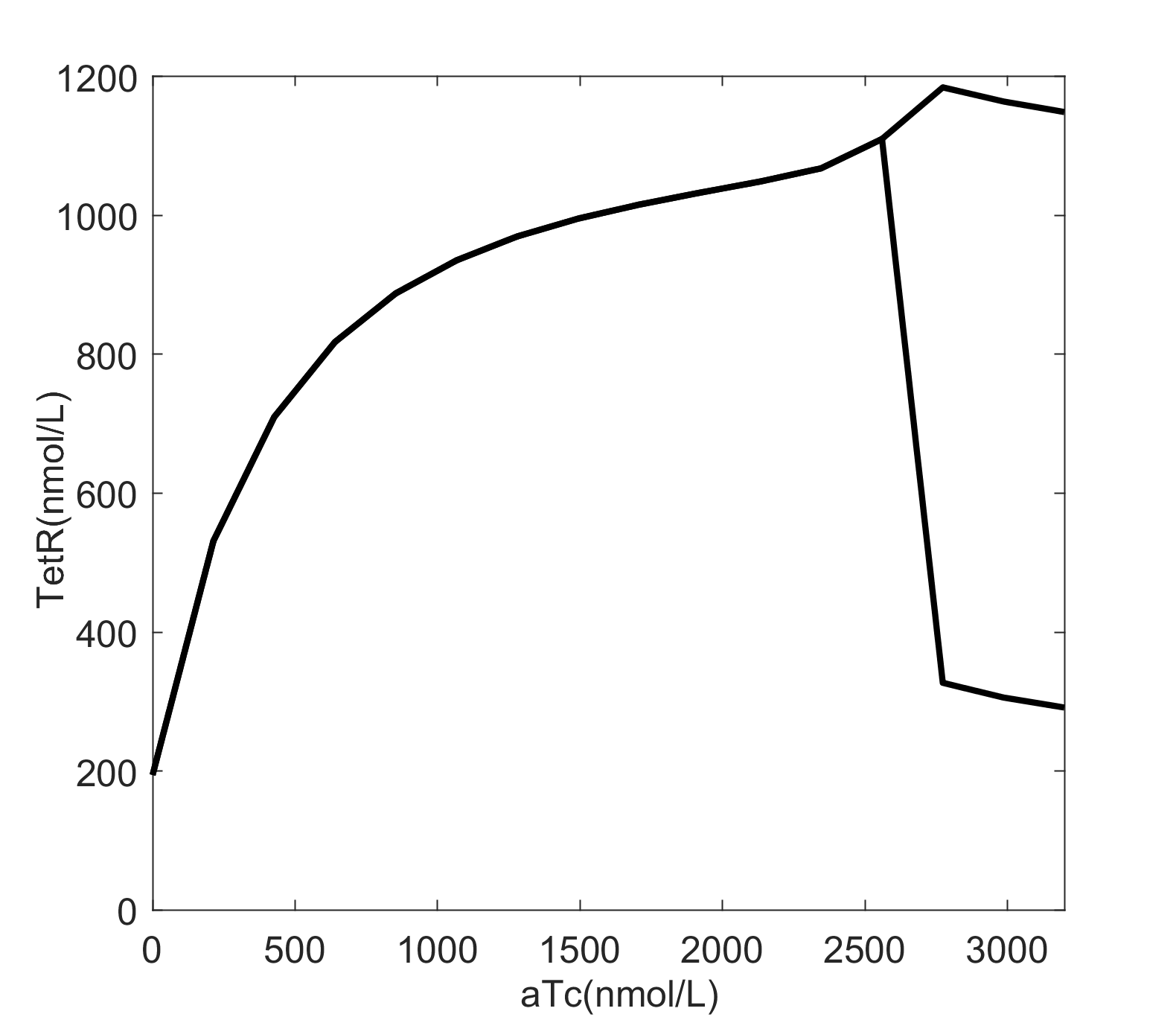


**Figure S13. The bifurcation diagram in total number of *TetR* (in nmol/L) molecules and aTc (in nmol/L) molecules.**


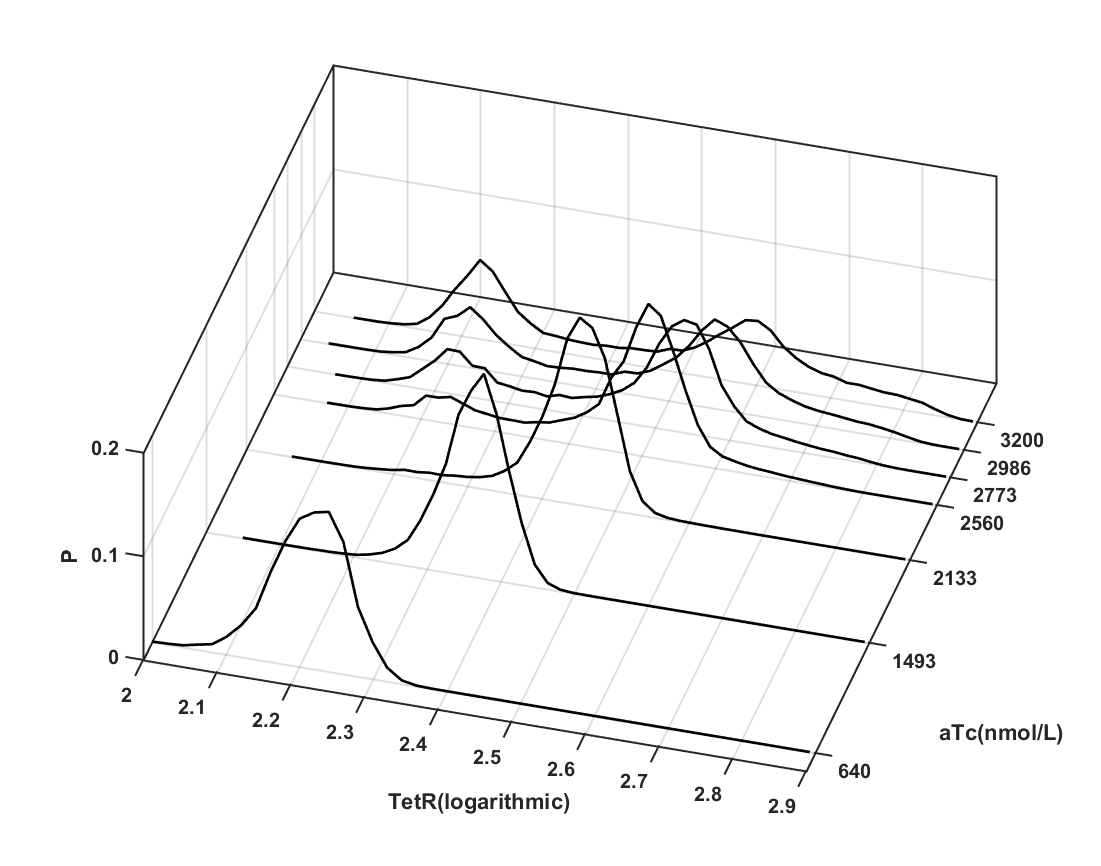


**Figure S14. The simulated steady state probability distribution in total *TetR* (in mol/L) and aTc (in mol/L ) molecules.**


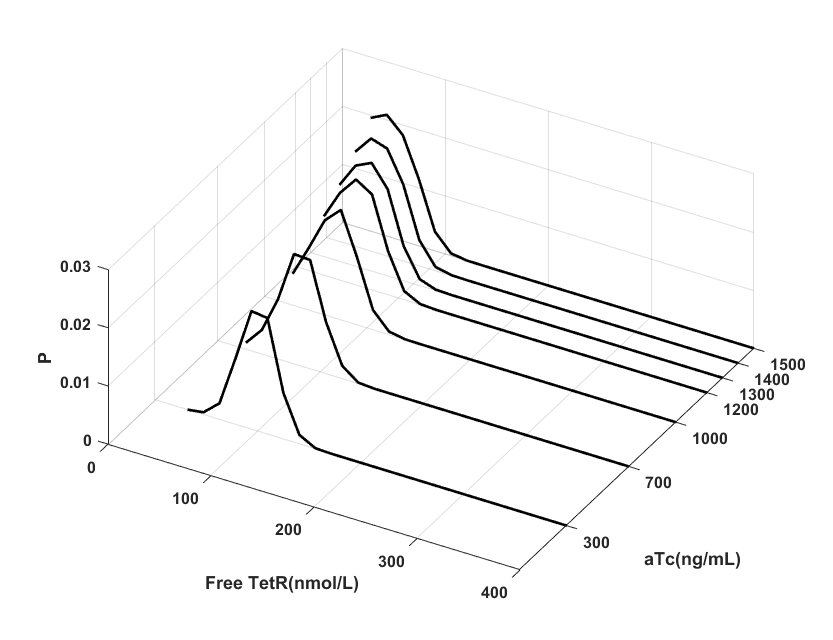


**Figure S15. The simulated steady state probability distribution in free *TetR* (in mol/L) and aTc (in mol/L ) molecules.**


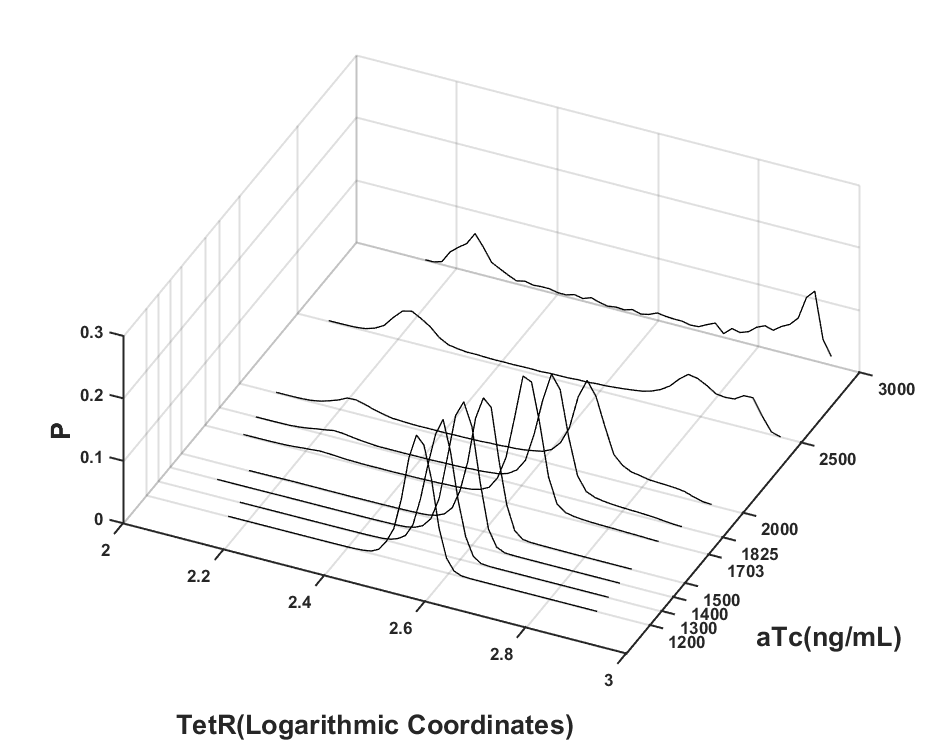


**Figure S16. The simulated steady state probability distribution with much higher aTc concentration when equilibrium constants f/h increases from 60 to 120.**


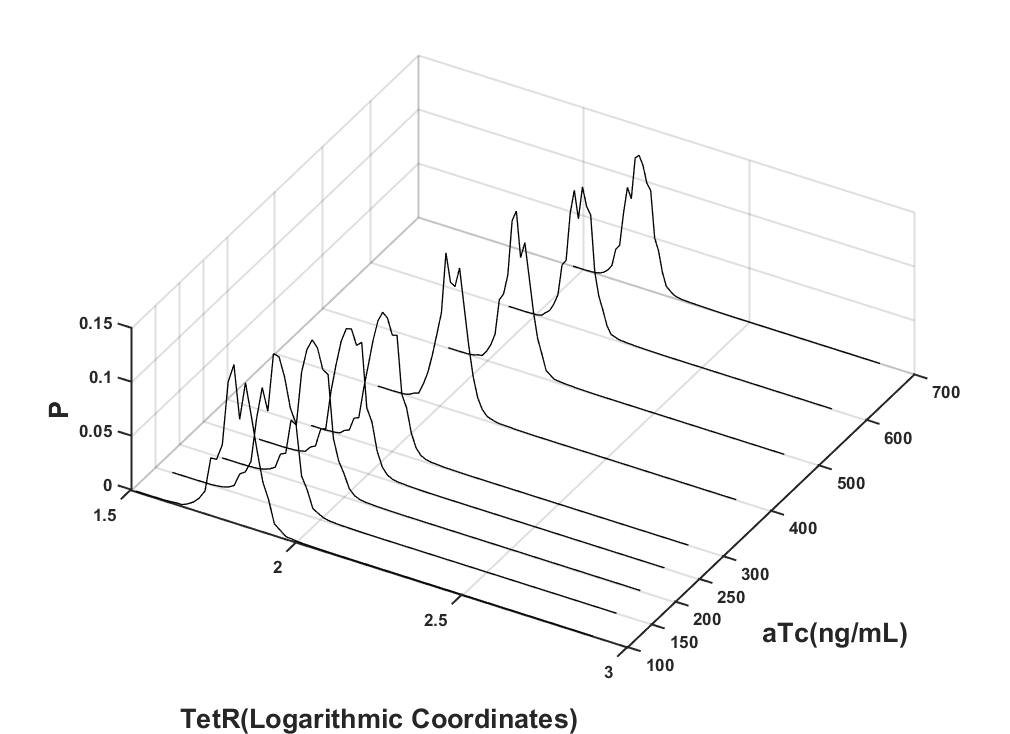


**Figure S17. The simulated steady state probability distribution in different aTc concentrations when equilibrium constants f/h decreases from 60 to 1.**


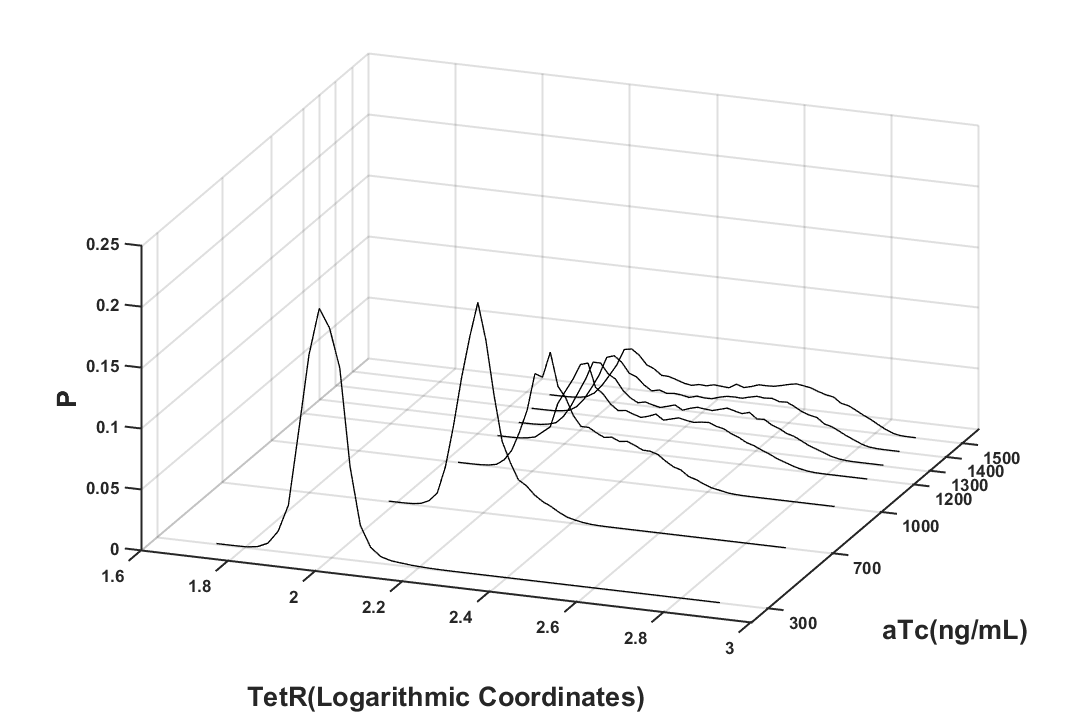


**Figure S18. The simulated steady state probability distribution in different aTc concentrations when the unbinding rate is assumed not to be concentration dependent.**
